# Plant‐Derived Melatonin Inhibits Bacterial Virulence via CpxA/R Two‐Component System

**DOI:** 10.1002/advs.74806

**Published:** 2026-03-12

**Authors:** Jin‐Wei Wei, Wei Liu, Bili Cao, Dan Zhao, Chengqiang Wang, Xin Hou, Tangyuan Ning, Biao Gong

**Affiliations:** ^1^ Shandong Agricultural University Taian China; ^2^ Institute of Genetics and Developmental Biology Chinese Academy of Sciences Beijing China

**Keywords:** CpxA, CpxR, melatonin, plant‐pathogen interaction, T3SS

## Abstract

In defending against pathogens, plants deploy diverse secondary metabolites and signaling molecules. Among these, melatonin orchestrates plant growth and development, modulates stress responses, and regulates intracellular redox homeostasis and signaling. However, the mechanisms of melatonin in plant‐pathogen interaction are rarely reported. Using *Pseudomonas syringae* pv. *tomato* DC3000 (*Pst* DC3000) as model bacteria, we designed a two‐step high‐throughput screening strategy to screen the plant natural product library and the bacterial mutant library. This study reveals that melatonin is perceived by a bacterial receptor histidine kinase CpxA, which subsequently modulates bacterial virulence. In detail, bacterial CpxA senses melatonin through Glu48 and Thr51 sites located in the periplasmic sensor region. Thus, melatonin inhibits autophosphorylation of CpxA and decreases transphosphorylation of the response regulator CpxR. The DNA‐binding capacity of CpxR to promoters of type III secretion system (T3SS) genes is weakened by reduced phosphorylation cascade of CpxA/R, inhibiting bacterial T3SS genes expression and virulence. We also showed that increasing melatonin synthesis in plants can enhance disease resistance and sustain crop productivity. This study illustrates a previously unknown mechanism by which plants disarm the pathogenicity of bacteria, as well as provide effective molecular targets for crop genetic improvement and biopesticides development.

## Graphical abstract

Plant‐derived melatonin is sensed by CpxA^E48/T51^, which inhibits the phosphorylation cascade transmission from CpxA^H240^ to CpxR^D52^, resulting in the inhibition of DNA‐binding capacity of CpxR and subsequent T3SS genes expression in *Pst* DC3000.

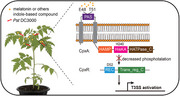

## 1 Introduction

The interaction between plants and pathogens operates through bidirectional, inter‐kingdom chemical communication. In this co‐evolutionary dialogue, plants detect pathogen‐associated molecular patterns (PAMPs) to activate immunity, while pathogens sense host signals to optimize virulence [1, 2]. Plant natural products are central to this exchange, functioning not only as direct antimicrobials but also as signaling molecules that precisely manipulate pathogen behavior [3, 4]. For instance, multiple defense compounds (e.g. erucamide, polyphenols, sulforaphane, and cytokinins) disarm the *Pseudomonas syringae* type III secretion system (T3SS) by targeting distinct components [5, 6, 7, 8]. Conversely, pathogens exploit host metabolites: *P. syringae* senses putrescine to upregulate T3SS, while others like *Ralstonia solanacearum* and *Xanthomonas campestris* utilize plant γ‐aminobutyric acid (GABA) and salicylic acid to promote infection [9, 10, 11]. Dissecting how plant metabolites influence disease is critical for developing novel crop protection strategies.

Two‐component system (TCS) is an evolutionarily highly conserved core environmental sensing and signal transduction mechanism in bacteria, mainly consisting of a transmembrane histidine kinase (HK) responsible for recognizing external signals and a cytoplasmic response regulator (RR) that mediates transcriptional regulation [12, 13, 14]. Upon sensing external stimuli including host signals and physicochemical stresses, the histidine kinase undergoes autophosphorylation and transfers a phosphate group to the response regulator; the activated response regulator can then bind to the promoters of target genes and regulate the expression of genes associated with bacterial virulence, colonization, drug resistance and metabolism [15, 16]. TCS serves as a key regulatory pathway for bacteria to adapt to the environment, infect hosts and exert pathogenicity, and its mechanism of action has been verified in a variety of plant and animal pathogens. In plant pathogens, polyphenols can target the histidine kinase RhpS and block the activation of the T3SS by inhibiting its phosphatase activity [8], while PcrK can specifically sense plant cytokinins to regulate oxidative stress responses and virulence expression [6]. Among animal pathogens, enterohemorrhagic *Escherichia coli* can sense microbiota‐derived nicotinamide to increase the phosphorylation level of EvgA [17], or sense host intestinal riboflavin via RbfS/R to enhance the phosphorylation of RbfS, thereby promoting virulence expression and intestinal colonization [18]. Overall, pathogens can precisely recognize key small molecules derived from hosts and commensal microbiota through diverse TCSs, thereby efficiently regulating virulence and colonization ability, which is an important strategy for them to adapt to the host environment and achieve infection and pathogenicity.

A central virulence node for many Gram‐negative plant pathogenic bacteria, including *P. syringae* pv. *tomato* DC3000 (*Pst* DC3000), is the T3SS. The T3SS is a needle‐like apparatus that injects effector proteins directly into host cells to suppress immunity and promote infection [19]. For instance: Plant erucamide disrupts bacterial type III injectisome assembly [5]; Plant cannabinoids inhibit bacterial QseC receptor to reduce T3SS function [20]. Conversely, certain plant‐derived metabolites, such as specific organic acids and amino acids, induce T3SS gene expression in pathogens [21]. Aspartic and glutamic acids, in particular, serve as potent chemotactic signals that promote bacterial motility and T3SS deployment [22]. Given the central role of the T3SS in bacterial virulence, identifying inter‐kingdom signals that modulate its function is a major research focus. Given its critical role, the T3SS is a prime target for plant immune interventions. Identifying plant compounds that selectively inhibit T3SS function, without exerting broad bactericidal pressure that drives resistance, is a key research frontier. Such anti‐virulence strategies could lead to sustainable disease management solutions.

Melatonin (N‐acetyl‐5‐methoxytryptamine) is a phylogenetically conserved molecule found in organisms ranging from bacteria to humans. In plants, melatonin is recognized not only as a potent antioxidant but also as a pleiotropic regulator of growth, development, and stress responses, leading to its classification as a phytohormone [23]. Its role in plant immunity is increasingly appreciated; melatonin enhances resistance to various pathogens, including *Pst* DC3000, often through modulating ROS homeostasis and potentiating defense hormone signaling pathways such as those of salicylic acid and jasmonic acid [24, 25]. Traditionally, the protective effects of melatonin have been attributed to its activities within the plant, including scavenging ROS, regulating gene expression, and strengthening cellular defenses.

However, an intriguing possibility remains largely unexplored: could melatonin, secreted by plants under attack, also function as an inter‐kingdom signal that is directly perceived by the invading pathogen? In animals, melatonin exerts its effects by binding to high‐affinity G‐protein coupled receptors MT_1_ and MT_2_ [26]. In plants, a membrane‐localized melatonin receptor (PMTR1/CAND2) that interacts with a Gα subunit has been identified [27]. Yet, no bacterial sensor or receptor for melatonin has been reported to date. Discovering whether and how bacteria perceive melatonin would fundamentally expand our understanding of this molecule's ecological and evolutionary role in host‐microbe interactions.

Here, we report that the bacterial histidine kinase CpxA functions as a specific sensor for plant‐derived melatonin. We demonstrate that melatonin binds to the periplasmic PAS domain of CpxA at residues Glu48 and Thr51, inhibiting its autophosphorylation and subsequent phosphotransfer to the response regulator CpxR. This attenuated CpxA/R phosphorylation cascade reduces the DNA‐binding affinity of CpxR for the promoters of core T3SS genes, thereby repressing their expression and diminishing bacterial virulence. Genetic evidence from both plant (melatonin‐deficient and overproducing genotypes) and bacterial (CpxA/R mutants and site‐specific sensing mutants) systems confirms that this pathway operates under physiological conditions during infection. Our study unveils a previously unknown layer of melatonin‐mediated defense, establishing a direct molecular link between a conserved host metabolite and bacterial virulence regulation. These findings provide a novel mechanistic model for inter‐kingdom signaling and highlight promising targets for engineering durable disease resistance and developing innovative biopesticides.

## 2 Results

### 2.1 Melatonin Inhibits Bacterial T3SS Genes Expression

**FIGURE 1:**
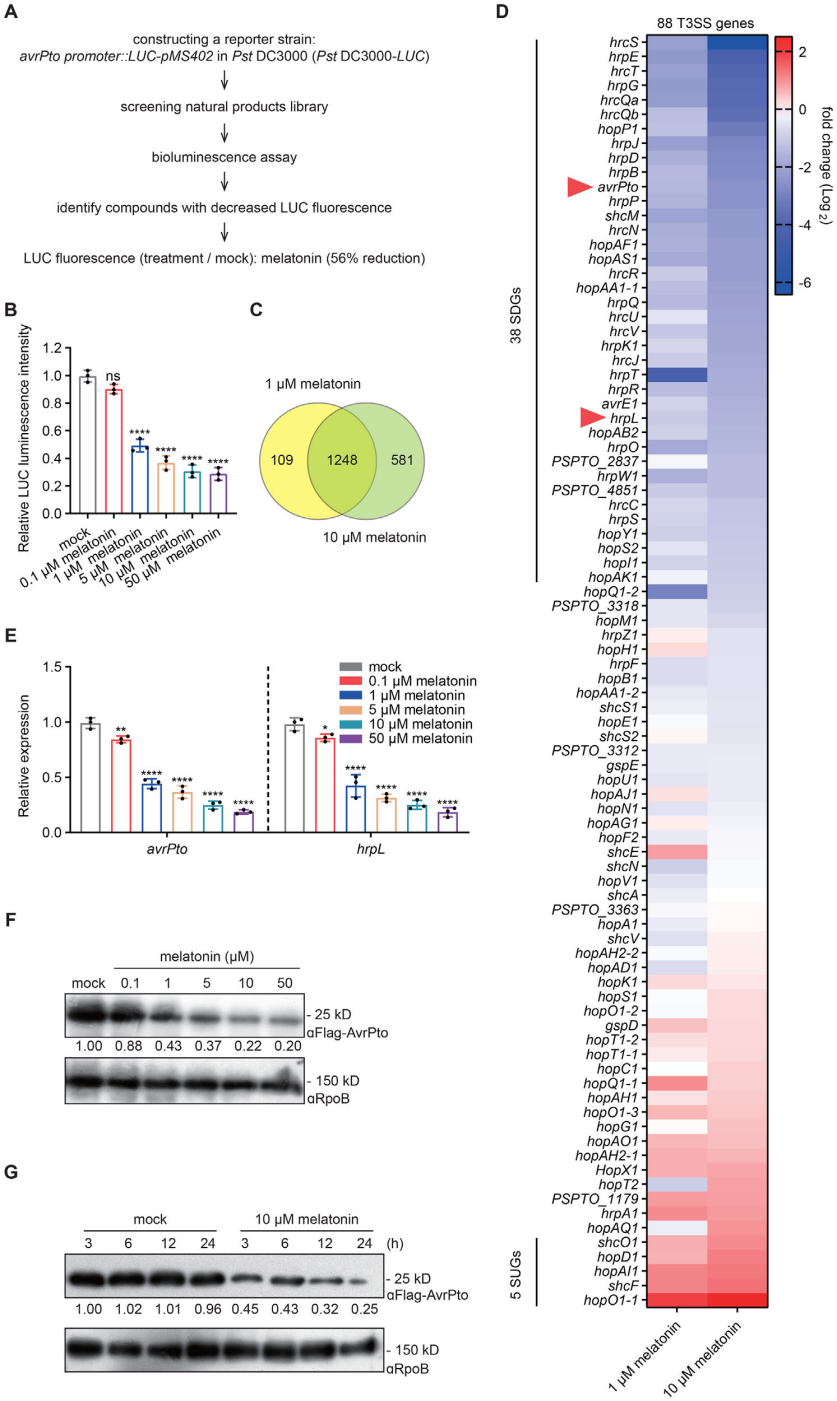
Melatonin inhibits bacterial T3SS genes expression. (A) Flow chart for high‐throughput screening of natural products that inhibit *Pst* DC3000 *avtPto* expression. The *Pst* DC3000‐*LUC* strain was cultured in King's B medium to OD_600_ = 0.6. Then, the cultures were washed three times by minimal medium (MM) liquid medium and diluted to an OD_600_ = 0.4. A commercial natural product library was screened to identify compounds with the capacity to inhibit *avrPto* expression. In brief, equal amount (50 µL) of fresh cultures were inoculated into each well of a 96‐well plate (black side wall and transparent bottom), which contained 50 µL MM liquid medium without (mock) or with 20 µM natural compounds in each well. After 12 h treatment, the treated cultures were incubated with 1 mM luciferin for 10 min. The LUC production was recorded by the GLO‐MAX 96 microplate luminometer. (B) Response of *avrPto* expression to melatonin. *Pst* DC3000‐*LUC* strain was incubated with 0–50 µM melatonin in MM liquid medium for 12 h, and then was used for LUC luminescence assay. Data represents means ± SD (n = 3; one‐way ANOVA with Dunnett's multiple comparison test; ^ns^ *P* > 0.05, ****P < 0.0001). (C) Venn diagram of differentially expressed genes (DEGs) in RNA‐Seq. (D) Expression pattern of 88 T3SS genes in RNA‐Seq. Gene expression levels for the 10 µM melatonin treatment group were sorted from low to high, revealing a set of 38 significantly down‐regulated genes (SDGs) and 5 significantly up‐regulated genes (SUGs). (E–G) Response of T3SS genes (*avrPto* and *hrpL*) and protein (AvrPto) to melatonin. *Pst* DC3000 was incubated without (mock) or with melatonin (0.1–50 µM for 12 h, or 10 µM for 3–24 h) in MM liquid medium, and then was used for RT‐qPCR and WB assays. Data of RT‐qPCR represents means ± SD (*n* = 3; one‐way ANOVA with Dunnett's multiple comparison test; *P < 0.05, **P < 0.01, ****P < 0.0001). Protein quantification was performed by ImageJ.

To screen plant natural products for their impact on bacterial virulence, we developed a T3SS reporter system by fusing the promoter region of the *avrPto* operon to the *luciferase* (*LUC*) gene in the *pMS402* vector. The resulting construct, *avrPto promoter::LUC‐pMS402*, was transformed into *Pst* DC3000 to create the reporter strain *Pst* DC3000‐*LUC*. Next, a commercial natural product library (Catalog No.L1400, Selleck Chem, USA) containing a total of 3,048 compounds was screened to identify compounds with the capacity to affect *avrPto* expression. The precultured *Pst* DC3000‐*LUC* strain was transferred to 96‐well plates containing minimal medium (MM) for virulence gene induction [28], followed by the addition of individual library compounds to a final concentration of 10 µM in each well. After 12 h treatment, relative LUC luminescence intensity of plates was measured. We focused on compounds that inhibited *avrPto* expression due to their potential relevance for plant disease resistance and biopesticide development. Screening results indicated that melatonin and several other natural products can inhibit the *avrPto* expression (Supplementary Datase). Melatonin is a molecule commonly found in both plants and bacteria, where it plays a key role in regulating redox balance—a crucial component of plant immunity. Therefore, it is important to investigate the molecular mechanism and direct target by which plant‐derived melatonin suppresses bacterial virulence. As shown, melatonin treatment suppressed the relative LUC signal intensity, reducing it to 44% of the control level (Figure 1A). A detectable decrease in *avrPto* expression was observed at 1 µM melatonin, and this inhibitory effect was enhanced in a concentration‐dependent manner from 1 to 50 µM (Figure 1B). We then assessed whether melatonin influenced bacterial growth, biofilm formation, or motility. None of these physiological traits were significantly affected by melatonin treatment, suggesting that melatonin specifically inhibits bacterial virulence without exerting bactericidal effects (Figure S1).

We conducted RNA sequencing (RNA‐Seq) on *Pst* DC3000 treated with 1 or 10 µM melatonin and a mock control. Relative to the control, 1 357 and 1 829 differentially expressed genes (DEGs) were identified at 1 and 10 µM melatonin, respectively, and 1 248 DEGs were shared between the two groups (Figure 1C). The magnitude of differential expression for most DEGs increased with melatonin concentration, as shown by hierarchical clustering analysis, supporting a dose‐dependent effect of melatonin on the transcriptome (Figure S2A). Kyoto Encyclopedia of Genes and Genomes (KEGG) analysis showed that the most up‐regulated genes by melatonin were enriched in core metabolic pathways, while the top down‐regulated pathways included ABC transporters, QS, and the two‐component system (Figure S3). The concerted suppression of these key virulence‐associated pathways indicates that melatonin likely inhibits bacterial virulence genes expression. This transcriptome profiled 291 out of 298 reported virulence genes, including 88 of 94 T3SS genes, in *Pst* DC3000 [29]. Treatment with 10 µM melatonin significantly altered the expression of many these genes, resulting in 33.68% (98/291) significantly down‐regulated genes (SDGs) and 16.15% (47/291) significantly up‐regulated genes (SUGs) among the virulence‐associated genes (Figure S2B). A more pronounced effect was observed specifically within T3SS genes, where 43.18% (38/88) were SDGs and only 5.68% (5/88) were SUGs (Figure 1D).

Given that *Pst* DC3000 requires a T3SS to inject virulence effectors like AvrPto into host cells [19], we selected the effector gene *avrPto* and the master regulator *hrpL* as representative markers to investigate the mechanism by which melatonin inhibits bacterial virulence. When *Pst* DC3000 was exposed to 0.1 µM melatonin, the expression of *avrPto* and *hrpL* was significantly inhibited, and the inhibitory effect was enhanced with the increase of melatonin concentrations (Figure 1E). Melatonin also inhibited the AvrPto protein accumulation in both dose‐dependent (0.1–50 µM, 12 h) and time‐dependent (10 µM, 3–24 h) manners (Figure 1F,G). Together, melatonin inhibits bacterial T3SS genes expression.

### 2.2 Bacterial CpxA is a Melatonin Sensor

**FIGURE 2:**
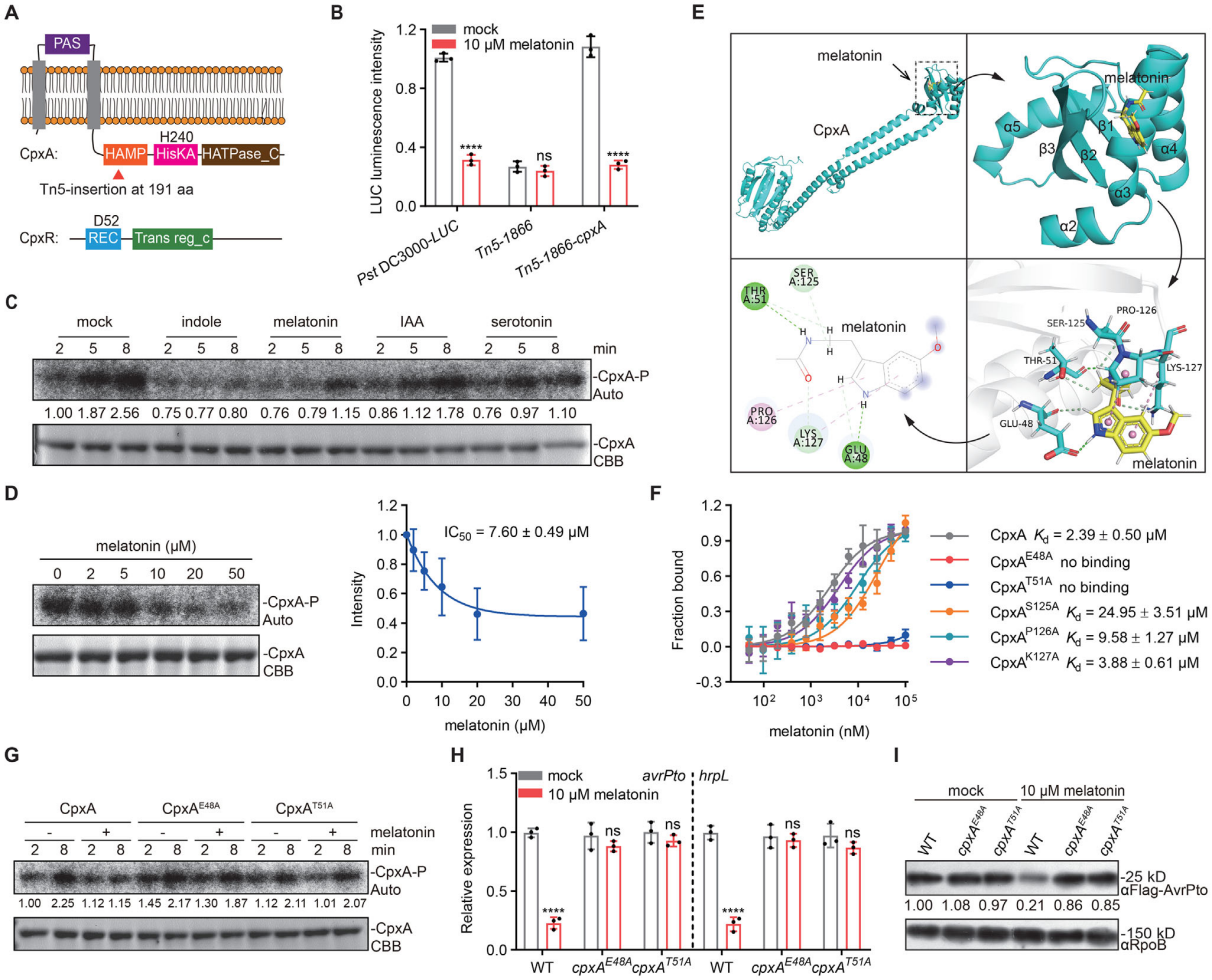
Bacterial CpxA is a melatonin sensor. (A) Character of CpxA and CpxR. The CpxA protein has a PAS (Per‐Arnt‐Sim) domain, HAMP (Histidine kinases, Adenylyl cyclases, Methyl binding proteins, Phosphatases) domain, HisKA [His Kinase A (phosphoacceptor)] domain and HATPase_C (Histidine kinase‐like ATPases) domain. The Tn5‐insertion site is located in 191 aa of HAMP domain. The CpxR protein has a REC (receiver) domain and Trans reg_c (transcriptional regulatory protein, C terminal) domain. (B) The *avrPto* expression. *Pst* DC3000‐*LUC*, *Tn5‐1866*, and complementary (*Tn5‐1866‐cpxA*) strains were incubated without or with 10 µM melatonin in minimal medium (MM) liquid medium for 12 h, and then was used for LUC luminescence assay. Data represents means ± SD (*n* = 3; two‐way ANOVA with Sidak's multiple comparison test; ^ns^ *P* > 0.05, ****P < 0.0001). (C) Autophosphorylation of CpxA. The CpxA protein was pretreated without (mock) or with 10 µM indole, melatonin, IAA, serotonin. Then, the autoradiography (Auto) was tested at 2, 5, 8 min after the addition of 10 µCi [γ‐^32^P]‐ATP. Coomassie brilliant blue (CBB) staining of CpxA served as loading control. Protein quantification was performed by ImageJ. (D) Dose‐dependent response of CpxA autophosphorylation to melatonin for calculation of IC_50_. The CpxA protein was pretreated with 0–50 µM melatonin and then the Auto was tested at 10 min after the addition of 10 µCi [γ‐^32^P]‐ATP. CBB staining of CpxA served as loading control. Protein quantification of was performed by ImageJ to calculate the IC_50_. Data represents means ± SD (*n* = 4). (E) Binding mode of melatonin with CpxA using molecular docking. (F) MST assay of interaction between CpxA/CpxA^E48A^/CpxA^T51A^/CpxA^S125A^/CpxA^P126A^/CpxA^K127A^ and melatonin. Data represents means ± SD (n = 3). (G) Autophosphorylation of CpxA, CpxA^E48A^, and CpxA^T51A^. Proteins were pretreated without or with 10 µM melatonin. Then, the Auto was tested at 2 and 8 min after the addition of 10 µCi [γ‐^32^P]‐ATP. CBB staining of CpxA served as loading control. Protein quantification was performed by ImageJ. (H,I) Response of genes (*avrPto* and *hrpL*) and protein (AvrPto) expression to melatonin. WT, *cpxA^E48A^*, and *cpxA^T51A^* strains were incubated without or with 10 µM melatonin in MM liquid medium for 12 h, and then were used for RT‐qPCR and WB assays. Data of RT‐qPCR represents means ± SD (*n* = 3; two‐way ANOVA with Sidak's multiple comparison test; ^ns^ *P* > 0.05, ****P < 0.0001). Protein quantification was performed by ImageJ.

To investigate the mechanism by which melatonin regulates T3SS genes expression, we conducted a screen using a Tn5‐insertion mutant library constructed in the *Pst* DC3000‐*LUC* background (Figure S4). Using previously established LUC reporter system with 10 µM melatonin, we screened approximately 10,000 Tn5‐insertion mutants for melatonin‐insensitive *avrPto* expression. This screen identified one mutant, *Tn5‐1866*, in which the Tn5‐insertion was located within a gene encoding a membrane‐associated histidine sensor kinase. The insertion caused premature translational termination at residue 191, resulting in a truncated protein that lacked part of the HAMP (Histidine kinases, Adenylyl cyclases, Methyl‐binding proteins, Phosphatases) domain and the entire HisKA [Histidine Kinase A (phosphoacceptor)] and HATPase_C (Histidine kinase‐like ATPases) domains (Figure 2A). The protein encoded by this gene showed high homology with the known histidine kinase CpxA and exhibited a high degree of conservation across the *Pseudomonas* genus (Figures S5–S7). We then retrieved CpxA protein sequences from 55 bacterial species from the KEGG database for evolutionary analysis, which revealed that the CpxA homolog in *Pst* DC3000 is most closely related to that of *Enterobacter cloacae* (Figure S8). So, we designated this gene *cpxA*, and generated a complemented strain (*Tn5‐1866‐cpxA*), in which melatonin‐dependent suppression of *avrPto* expression was restored (Figure 2B).

Melatonin is an indole‐based compound with similar chemical structure to indole and serotonin. In enterohemorrhagic *Escherichia coli*, indole and serotonin have been evidenced to inhibit bacterial virulence via CpxA [30, 31]. Since CpxA contains a PAS (Per‐Arnt‐Sim) domain in periplasmic sensor region [32], we speculated that CpxA may be a melatonin sensor, which senses melatonin and alters its autophosphorylation state and kinase activity. Using indole, serotonin, and indoleacetic acid (IAA) as control, we found that melatonin and indole had similar inhibitory effects on the autophosphorylation of CpxA; however, the inhibition degree of serotonin and IAA were weaker than that of melatonin (Figure 2C). Among the compounds tested in *Pst* DC3000, indole exerted the strongest inhibition on *avrPto* and *hrpL* expression, followed by melatonin, while serotonin and IAA had minimal effects (Figure S9A,B). Further, the half maximal inhibitory concentration (IC_50_) of melatonin to CpxA was calculated as 7.60 ± 0.49 µM according to a dose‐dependent response of CpxA autophosphorylation to melatonin treatment (Figure 2D).

In molecular docking (Figure 2E), the CpxA‐melatonin interaction region is located in the periplasmic PAS domain of CpxA with a typical melatonin‐binding pocket structure. More finely, the Glu48 (E48), Thr51 (T51), and Ser125 (S125) sites of the CpxA interact with melatonin in the form of hydrogen bonds; the Pro126 (P126) and Lys127 (K127) sites participate in this interaction in the form of van der Waals forces. To determine the conclusion of molecular docking, MicroScale Thermophoresis (MST) assay was performed to show a strong interaction between CpxA and melatonin with a dissociation constant (*K*_d_) value of 2.39 ± 0.50 µM (Figure 2F). Similarly, melatonin analogues, including indole, serotonin, and IAA, also directly interacted with CpxA with micromolar *K*_d_ values (Figure S9C–E). When E48, T51, S125, P126, and K127 sites of CpxA were respectively mutated into Ala (A), the CpxA^E48A^ and CpxA^T51A^ mutant proteins lost their binding ability to melatonin; however, the CpxA^S125A^, CpxA^P126A^, and CpxA^K127A^ proteins had little effect on its interaction with melatonin (Figure 2F).

We performed the autophosphorylation response of CpxA, CpxA^E48A^, and CpxA^T51A^ to melatonin, showing that both CpxA^E48A^ and CpxA^T51A^ mutants abolished the melatonin‐inhibited autophosphorylation (Figure 2G). We constructed the *cpxA* ^E48A^ and *cpxA* ^T51A^ strains using site‐directed mutagenesis and analyzed the response of *avrPto* and *hrpL* expression, and AvrPto protein accumulation to melatonin. Compared to the WT strain, both *cpxA^E48A^* and *cpxA^T51A^* strains were insensitive to melatonin (Figure 2H,I). Together, CpxA is a melatonin sensor in *Pst* DC3000.

### 2.3 Melatonin Inhibits CpxA/R Phosphorylation Cascade to Drive T3SS Transcriptional Reprogramming

**FIGURE 3:**
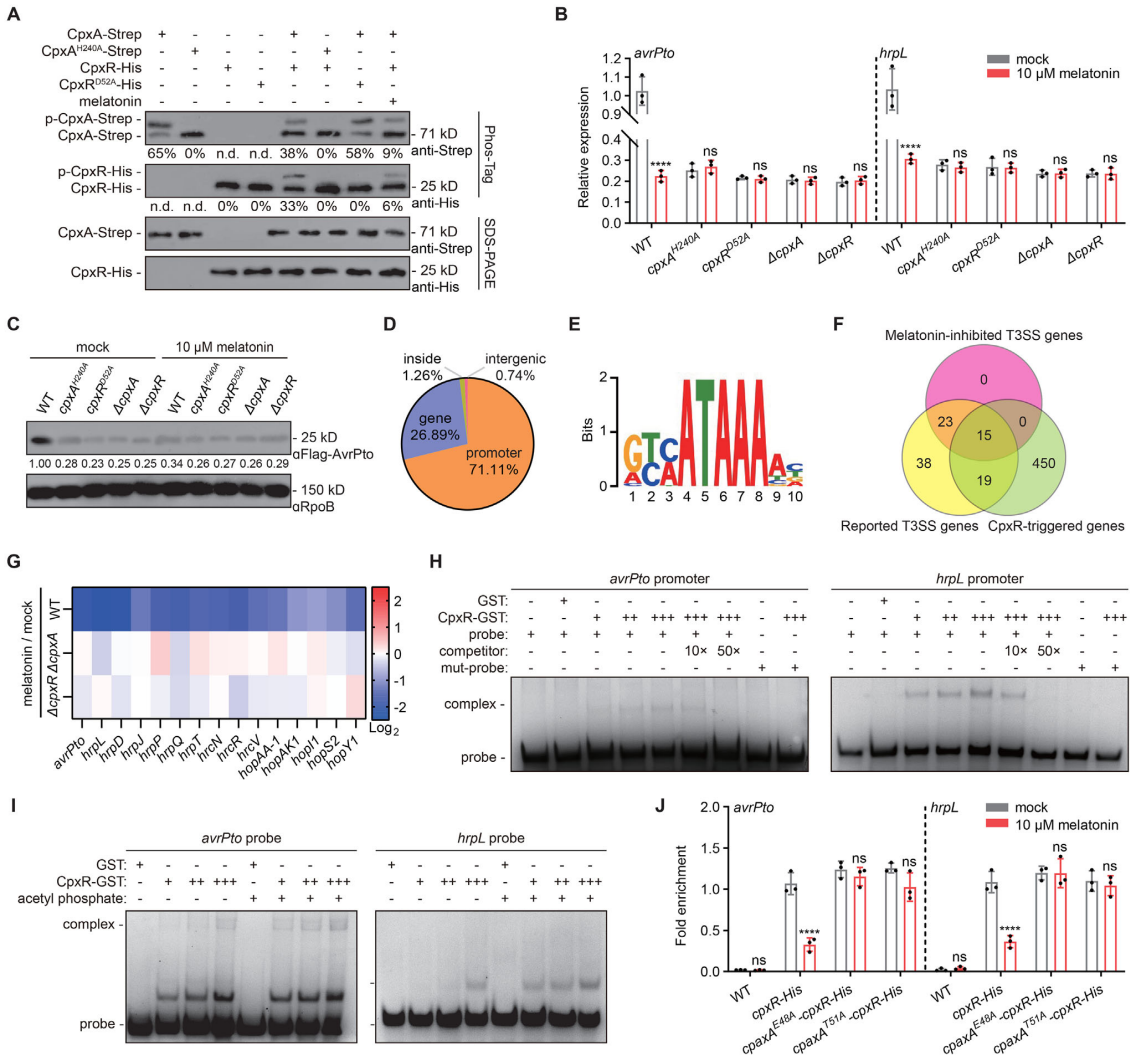
Melatonin inhibits CpxA/R phosphorylation cascade to drive T3SS transcriptional reprogramming. (A) In vitro kinase assay shows that melatonin inhibits CpxA/R phosphorylation cascade. Recombinant proteins CpxA‐Strep (wild‐type or H240A mutant) were incubated with CpxR‐His (wild‐type or D52A mutant) in kinase assay buffer to analyze autophosphorylation and phosphotransfer. The effect of 10 µM melatonin on the phosphotransfer from CpxA to CpxR was assessed. Protein levels and phosphorylation status were monitored by Phos‐Tag assay with SDS‐PAGE as input. Protein quantification was performed by ImageJ. (B,C) Response of T3SS genes (*avrPto* and *hrpL*) and AvrPto protein expression to melatonin. WT, *cpxA^H240A^*, *cpxR^D52A^*, *ΔcpxA*, and *ΔcpxR* strains were incubated without or with 10 µM melatonin in minimal medium (MM) liquid medium for 12 h, and then were used for RT‐qPCR assay and WB assays. Data of RT‐qPCR represents means ± SD (*n* = 3; two‐way ANOVA with Sidak's multiple comparison test; ^ns^ *P* > 0.05, ****P < 0.0001). Protein quantification was performed by ImageJ. (D) Genome‐wide distribution analysis of the CpxR‐binding sites in ChIP‐Seq. (E) Binding motifs of CpxR in genome promoter regions in ChIP‐Seq. (F) Venn diagram of reported T3SS genes [29], melatonin‐inhibited T3SS genes (Figure 1d), and CpxR‐triggered genes (promoter regions with CpxR‐binding sites in ChIP‐Seq). (G) Melatonin inhibits the expression of core T3SS genes (intersection of Venn diagram) via the CpxA/R system. WT, *ΔcpxA*, and *ΔcpxR* strains were incubated without or with 10 µM melatonin in MM liquid medium for 12 h, and then were used for RT‐qPCR assay. The heatmap depicts the fold‐change in gene expression (melatonin / mock) for each strain, revealing that the deletion of *cpxA* or *cpxR* abolishes melatonin's inhibitory effect. Complete data are presented in Figure S13. (H) EMSA shows direct binding of CpxR to *avrPto* and *hrpL* promoter probes. Recombinant CpxR‐GST protein was incubated with 5’‐FAM‐labeled promoter probes. Negative controls included probe alone, addition of unlabeled probe (competitor), and mutant probe. (I) Phosphorylation enhances DNA‐binding capacity of CpxR to promoters of *avrPto* and *hrpL*. Different concentrations of CpxR‐GST recombinant protein were pretreated without or with acetyl phosphate, and then were used for EMSA with 5’‐FAM‐labeled probe (*avrPto* or *hrpL* probe). Negative control: probe with GST recombinant protein. (J) ChIP‐qPCR assay. WT, *cpxR‐His*, *cpxA^E48A^‐His*, and *cpxA^T51A^‐His* strains were incubated without or with 10 µM melatonin in MM liquid medium for 12 h, and then were used for ChIP‐qPCR assay. Data represents means ± SD (n = 3; two‐way ANOVA with Sidak's multiple comparison test; ^ns^ *P* > 0.05, ****P < 0.0001).

Two‐component system is conserved in bacteria to sense multiple stimulus and adapted to variable stress [30, 31, 33]. A CpxA/R two‐component system is composed by the transmembrane sensor kinase CpxA and the cytoplasmic response regulator CpxR [34]. Based on the reported bacterial CpxR protein sequence, we identified a putative *Pst* DC3000 CpxR (Figures S10 and S11). The CpxR has a REC (receiver) domain and Trans reg_c (transcriptional regulatory protein, C terminal) domain (Figure 2A). The REC domain contains the phosphoacceptor site for histidine kinases, whereas the associated Trans_reg_c domain is responsible for DNA binding. In response to extracytoplasmic stress, CpxA autophosphorylates at His240 (H240) and transfers the phosphate to CpxR at Asp52 (D52), thereby allowing CpxR to control the transcription of downstream genes ([35, 36]; Figure 2A). We systematically investigated the CpxA/R phosphorylation cascade in *Pst* DC3000, defining the key phosphorylation events and their regulation by melatonin. Figure 3A demonstrates that CpxA autophosphorylates at H240 (lanes 1,2) and that this site is essential for phosphotransfer to CpxR (lanes 1–6); D52 acts as the specific phosphoacceptor site in CpxR (lanes 1–7). Having established this, we together showed that melatonin binds to CpxA (Figure 2F), inhibits its autokinase activity (Figure 2D), and consequently blocks the phosphotransfer to CpxR (Figure 3A, lanes 5,8). To determine whether melatonin inhibits T3SS genes expression via the CpxA/R phosphorylation cascade, we constructed deletion mutants (*ΔcpxA* and *ΔcpxR*) and site‐specific mutants (*cpxA^H240A^* and *cpxR^D52A^*). Under mock‐treated conditions, all mutants exhibited significantly reduced expression of *avrPto* and *hrpL*, as well as lower AvrPto protein abundance, compared to the WT (Figure 3B,C). This confirms that the CpxA/R phosphorylation cascade is intrinsically required for T3SS activation. Notably, melatonin treatment suppressed T3SS genes expression in the WT but failed to do so in any of the mutant backgrounds (Figure 3B,C). Together, these results demonstrate that a functional CpxA/R phosphorylation cascade is essential for melatonin‐mediated inhibition of T3SS genes expression.

To define the direct regulatory role of CpxR on T3SS genes, we combined chromatin immunoprecipitation sequencing (ChIP‐Seq), electrophoretic mobility shift assays (EMSA), and ChIP‐qPCR analyses. ChIP‐Seq revealed that CpxR‐binding sites are highly enriched in promoter regions and contain a conserved ATAAA motif (71.11% of peaks; Figure 3D,E). This motif was present in the *avrPto* and *hrpL* promoters (Figure S12), and EMSA confirmed that CpxR binds directly to these regions in a specific and concentration‐dependent manner—a interaction abolished by mutation (ATAAA to CGCCC) of the motif (Figure 3H). The in vivo relevance was verified by ChIP‐qPCR, showing significant promoter enrichment in a *cpxR*‐His strain (only see WT and *cpxR*‐His strains in mock treatment; Figure 3J). Thus, we identify the ATAAA motif as the critical DNA recognition site for CpxR and essential for its direct control of T3SS genes. By integrating previously reported T3SS genes [29] with those inhibited by melatonin (Figure 1D) and those identified as CpxR targets, we identified a core set of 15 T3SS genes (Figure 3F). We therefore define these 15 genes as the key T3SS genes that are specifically and directly regulated by melatonin through the CpxA/R pathway. Gene expression analysis revealed that melatonin significantly suppressed the expression of these 15 T3SS genes in the WT strain, but this suppression was abolished in both *ΔcpxA* and *ΔcpxR* mutants, demonstrating that the inhibitory effect of melatonin depends entirely on a CpxA/R pathway (Figure 3G; Figure S13). Given that melatonin suppresses *avrPto* and *hrpL* expression via the CpxA/R pathway, we proposed that CpxR phosphorylation enhances its DNA binding. This was confirmed by EMSA, which showed stronger DNA binding by phosphorylated CpxR (Figure 3I). Consistent with this, ChIP‐qPCR assays revealed that melatonin reduces promoter enrichment in a *cpxR‐His* strain but not in *cpxA^E48A^‐His* and *cpxA^T51A^‐His* mutants (Figure 3J). Collectively, our results demonstrate that melatonin inhibits T3SS genes expression by attenuating the CpxA/R phosphorylation cascade and the subsequent DNA binding of CpxR.

### 2.4 Plant‐Derived Melatonin Confers Persistent Resistance and Crop Productivity

**FIGURE 4:**
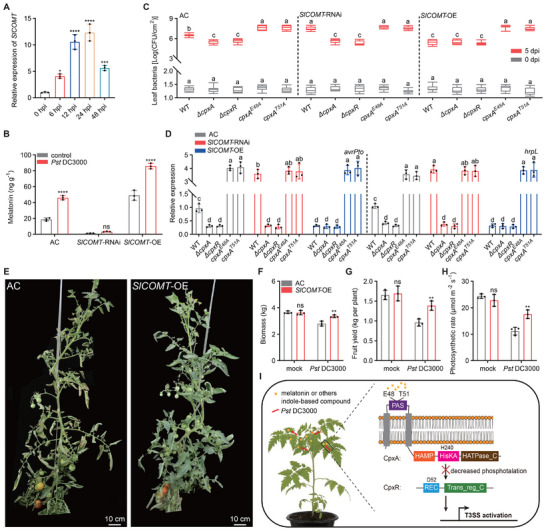
Plant‐derived melatonin confers persistent resistance and crop productivity. (A) Transcriptional response of *SlCOMT* to *Pst* DC3000 infection. AC tomato plants were sprayed with *Pst* DC3000 suspension (10^7^ CFU mL^−1^). Leaves were harvested for RT‐qPCR assay at 0–48 hpi. Data represents means ± SD (*n* = 3; one‐way ANOVA with Dunnett's multiple comparison test; *P < 0.05, ***P < 0.001, ****P < 0.0001). (B) Melatonin contents. AC, *SlCOMT*‐RNAi, and *SlCOMT*‐OE tomato plants were sprayed without or with *Pst* DC3000 suspension (10^7^ CFU mL^−1^). Leaves were harvested for melatonin measurement at 24 hpi. Data represents means ± SD (*n* = 3; two‐way ANOVA with Sidak's multiple comparison test; ^ns^ *P* > 0.05, ****P < 0.0001). (C,D) Bacterial growth and T3SS genes (*avrPto* and *hrpL*) expression in tomato leaves. AC, *SlCOMT*‐RNAi, and *SlCOMT*‐OE tomato plants were sprayed with bacterial suspension of WT, *ΔcpxA*, *ΔcpxR*, *cpxA^E48A^*, and *cpxA^T51A^* strains (10^7^ CFU mL^−1^). Bacterial growth was measured at 0 and 5 dpi; boxplots show median with 0.25 and 0.75 quartiles, whiskers represent values from minimum to maximum (*n* = 8, one‐way ANOVA with Dunnett's multiple comparison test; different letters indicate significant differences at *P* < 0.05). RT‐qPCR were performed at 3 dpi; data represents means ± SD (*n* = 3; one‐way ANOVA with Dunnett's multiple comparison test; different letters indicate significant differences at *P* < 0.05). (E–H) Resistance phenotype (scale bar = 10 cm), biomass, fruit yield, and photosynthetic rate of AC and *SlCOMT*‐OE plants in field conditions. Plants were sprayed with *Pst* DC3000 suspension (10^7^ CFU mL^−1^) at 30, 60, and 90 days after planting. Indexes were measured at 130–150 d after planting. Data represents means ± SD (*n* = 3; two‐way ANOVA with Sidak's multiple comparison test; ^ns^ *P* > 0.05, **P < 0.01). (I) Mechanism model. Plant‐derived melatonin is sensed by CpxA^E48/T51^, which inhibits the phosphorylation cascade transmission from CpxA^H240^ to CpxR^D52^, resulting in the inhibition of DNA‐binding capacity of CpxR and subsequent T3SS genes expression.

To determine whether melatonin synthesis is a natural plant defense mechanism, we measured changes in the key gene *caffeic acid O‐methyltransferase* (*SlCOMT*) and melatonin levels in wild‐type Ailsa Craig (AC) tomato (*Solanum lycopersicum*) plants infected with *Pst* DC3000 [37, 38]. The transcript levels of *SlCOMT* were up‐regulated following *Pst* DC3000 infection, accompanied by increased melatonin accumulation (Figure 4A,B). We then created *SlCOMT* RNA‐interference (*SlCOMT*‐RNAi) and *SlCOMT* overexpression (*SlCOMT*‐OE) plants, which show altered melatonin levels, particularly upon *Pst* DC3000 infection (Figure 4B). The AC, *SlCOMT*‐RNAi, and *SlCOMT*‐OE plants were inoculated with the WT, *ΔcpxA*, *ΔcpxR*, *cpxA^E48A^*, and *cpxA^T51A^* strains for bacterial *in planta* growth and T3SS genes (*avrPto* and *hrpL*) expression assays (Figure 4C,D). First, the WT *Pst* DC3000 showed stronger bacterial growth and higher T3SS gene expression in *SlCOMT*‐RNAi plants, but weaker in *SlCOMT*‐OE plants, indicating that endogenous melatonin levels in tomato negatively regulate bacterial pathogenicity. However, in AC, *SlCOMT*‐RNAi, and *SlCOMT*‐OE backgrounds, both *ΔcpxA* and *ΔcpxR* mutants exhibited significantly reduced bacterial growth and T3SS gene expression compared to the WT strain, demonstrating that mutation of *cpxA* or *cpxR* impairs bacterial virulence. Notably, in the AC background, the *cpxA^E48A^* and *cpxA^T51A^* mutants displayed significantly higher bacterial growth and T3SS gene expression than the WT strain, and these measures were unaffected by changes in melatonin, suggesting that melatonin exerts its function primarily through the E48 and T51 sites of CpxA. To consolidate these findings, the key melatonin synthesis gene (*serotonin N‐acetyltransferase*, *AtSNAT*, [25]) expression, bacterial growth and T3SS gene expression were measured in Columbia (Col‐0) and *AtSNAT*‐knockout (*atsnat*) mutants inoculated with the WT and various mutant *Pst* DC3000 strains; similar phenotypes to those observed in tomato were identified (Figure S14).

To identify roles of melatonin in tomato sustained resistance under field conditions, we tested the *Pst* DC3000 resistance, plant growth, yield, and photosynthesis of AC and *SlCOMT*‐overexpression (*SlCOMT*‐OE) plants during the whole growth period under real production conditions (Figure 4E–H). Little difference of growth state, yield, and photosynthesis between AC and *SlCOMT*‐OE plants were observed under normal growth conditions, indicating that melatonin accumulation does not cause adverse agronomic traits. Under *Pst* DC3000 infectious state, *SlCOMT*‐OE plants acquired a stronger disease‐resistant phenotype than AC plants, including less leaf yellowing and disease spots, as well as higher biomass, yield, and photosynthesis. In terms of yield, pathogen infection resulted in 42.05% reduction in AC plants, but only 18.11% reduction in *SlCOMT*‐OE plants.

## 3 Discussion

Melatonin, with primary function of antioxidant and secondarily‐evolved function of signaling molecule, has been detected in almost all organisms, including animals, plants, bacteria, fungi, macroalgae, and protists [39]. In animals, the melatonin receptors are classified into MT_1_, MT_2_, and MT_3_ subtypes, among which MT_1_ and MT_2_ are high‐affinity G‐protein‐coupled receptors and MT_3_ is a quinone reductase 2 [26]. Plant melatonin receptor is a plasma membrane‐localized receptor‐like topology that interacts with the G‐protein ɑ subunit [27, 40]. In *Xanthomonas oryzae*, melatonin inhibits T3SS gene expression through the transcriptional regulator HpaR1 [41]. In *Magnaporthe oryzae*, melatonin directly binds to MoIcl1, that is also the target of fungicide isoprothiolane, and functions synergistically to increase the effect of fungicide [42]. However, whether there are melatonin receptors or sensors in bacteria has not been reported. Due to the absence of G‐protein‐coupled signaling elements in bacteria, we conjecture that the bacterial melatonin signaling pathway is likely to be different from the established signaling patterns in animals and plants. This study highlights that melatonin, as a ligand, is recognized by CpxA, specifically inhibits the phosphorylation cascade of CpxA/R two‐component system and subsequent T3SS genes expression. Although CpxA has a certain affinity for melatonin, and this binding alters the autophosphorylation of CpxA and its cascade transmission, resulting in physiological response processes affecting the function of T3SS. But we still consider defining CpxA as a melatonin sensor rather than a receptor. This is mainly based on the specificity of receptor in ligand recognition, subcellular localization and evolutionary homology. First, CpxA can also bind to the analogues of melatonin, which means nonspecific structural complementarity between CpxA and melatonin. Second, the PAS domain of CpxA is located in periplasm and senses the host‐derived melatonin in the infectious state. This is thought to be a bacterial response to environmental melatonin, rather than self‐produced melatonin. Melatonin synthesis in bacteria is not limited by tryptophan and can start with *D*‐erythrose‐*4*‐phosphate and phosphoenolpyruvate [39]. Although we can not construct melatonin‐deficient bacteria, it is still an interesting topic to investigate whether the bacteria's own melatonin acts on the CpxA/R two‐component system. Based on the conservative function of melatonin in regulating ROS homeostasis, we speculate that co‐evolution of host and pathogen may have formed a compromise mechanism using melatonin‐CpxA/R module to reduce T3SS function.

Melatonin is an indole compound which is structural similarity to indole and its derivatives [23]. Indole substances are widely found in natural products and drugs. Due to the unique structure and properties, these compounds exhibit distinctive physiological activities and have led to widespread attention in the field of pesticide development [43]. In drug discovery, indole is one of the key structural motifs and serves as a scaffold for a variety of receptor ligands [44]. Therefore, MST assays not only shows the binding of CpxA to melatonin, but also some other indole compounds. This view is supported by CpxA interactions with indole and serotonin in animal pathogenic bacteria [30, 31, 45]. Compared with previous studies, we further identified the fine site of CpxA's recognition of melatonin. From the perspective of structural biology and biochemistry, we speculate that plants use the melatonin binding property of CpxA^E48/T51^ to inhibit bacterial virulence. Indole derivatives exhibit unique properties such as mimicking peptide structures and reversible binding to enzymes, transcription factor and others [43]. Indole and serotonin can be used to modulate *C. rodentium* disease by regulating CpxA too [30, 31, 46]. Now, we have expanded the recognition of melatonin by CpxA, which has strongly promoted the potential application of indole compounds in improving crop resistance and developing biopesticides.

Since melatonin can be synthesized in both plants and bacteria, the biological significance of extracellular melatonin perception by bacteria is worth pondering. First, the periplasmic PAS domain of CpxA has the binding capacity of melatonin. Second, the melatonin‐CpxA/R signaling module specifically inhibits T3SS genes expression and virulence without affecting bacterial growth. Third, the biological function of melatonin is mainly antioxidant. These reasons let us to speculate that pathogenic bacteria, especially biotrophic bacteria like *Pst* DC3000, may need such recognition mechanism to sense the REDOX state and immune strength of host plants. It is well known that the process by which pathogenic bacteria increase virulence requires a lot of energy, inhibiting bacterial growth and reproduction. Therefore, the expression of virulence genes tends to peak at the initial stage of infection. Once successfully colonized, the bacteria reduce virulence and promote growth and reproduction. Plants usually have a lower oxidation state when their melatonin level is elevated ([39, 47] and 2019). It is possible that bacteria have developed a mechanism to recognize the “safety signal”, such as melatonin, during long‐term co‐evolution with the host plants to balance the energy allocation between virulence and growth processes. Therefore, we can define melatonin as a QS or inter‐kingdom signaling molecule. Plants have also formed disease resistance mechanism that use melatonin as a disguised “safety signal” to inhibit bacterial virulence and infection. In addition to REDOX state, other immune signaling pathways such as salicylic acid and jasmonic acid regulated by melatonin are also involved in disease resistance [24, 48]. In conclusion, plant‐derived melatonin can be sensed by bacterial CpxA and its cascade CpxR, which inhibits bacterial T3SS genes expression and virulence. These findings suggest a multi‐layered melatonin‐based arms race between plants and pathogenic bacteria. However, the role of melatonin in regulating plant immunity should not be overlooked. For example, mutation of the plant melatonin receptor leads to loss of resistance against *Pst* DC3000 [49], indicating that melatonin signaling plays a crucial role in disease resistance. However, prior research has largely depended on exogenous melatonin treatment, which diverges from the natural context of plant‐pathogen interactions. Our strategy of genetically modulating endogenous melatonin aims to preserve this native physiological state.

A critical limitation lies in our use of 10 µM melatonin in vitro, a concentration higher than plant's physiological range. Melatonin levels of *Arabidopsis* [50] and tomato [37, 48] remain within the nanomolar range; even with overexpression of key melatonin synthesis genes, physiological concentrations typically increase only severalfold. This significant discrepancy warrants explicit discussion regarding its biological relevance. Melatonin, with strong reducing properties, is light‐sensitive and prone to oxidative degradation or photodegradation in vitro. This results in its effective concentration in vitro often being significantly higher than physiological concentrations observed in vivo. In numerous botanical studies, exogenous melatonin is commonly applied at concentrations ranging from 50 to 200 µM, far exceeding its physiological levels ([3] and 2019; [37, 40, 48]). This suggests that melatonin's reactive chemical properties, transmembrane transport efficiency, and differences between synthetic melatonin and natural melatonin (e.g., molecular chirality) may be key factors contributing to the discrepancy in effective concentrations between in vitro and in vivo conditions. Consequently, the use of micromolar concentrations has become common practice and is considered biologically relevant in this field of research. Similar phenomenon is also common in studies of other phytohormones. For example, endogenous strigolactone levels in plants are picomolar; in contrast, exogenous applications often require concentrations as high as 5 µM to elicit responses in plants and microorganisms (Wang et al., 2020a; [51]). Bacterial PcrK senses plant cytokinins (picomolar level), while bacterial PcrK senses micromolar level cytokinins in vitro experiments [6]. The present study provided the bidirectional genetic studies using melatonin‐deficient plants and *CpxA/R*‐impaired bacteria demonstrated that CpxA can indeed perceive melatonin fluctuations and regulate bacterial T3SS genes expression and pathogenicity. This indicates that melatonin at physiological concentrations can functionally regulate the CpxA/R‐T3SS module in vivo. Notably, we must acknowledge the heterogeneous distribution of melatonin in plant tissues—for instance, chloroplasts may exhibit higher melatonin levels than the cytoplasm by analyzing the subcellular localizations of melatonin synthase [52]. A hypothesis thus arises that localized melatonin concentrations at plant‐pathogen interaction interfaces may be enriched to micromolar levels; however, current study lacks experimental schemes to validate this conjecture. In the context of biopesticides development and target research, exogenous application of micromolar melatonin effectively suppresses bacterial T3SS genes expression and virulence. Regarding genetic engineering for crop improvement, we have demonstrated that manipulating melatonin synthesis enhances plant disease resistance while inhibiting bacterial T3SS genes expression and pathogenicity.

## 4 Conclusion

This study demonstrates that plant indeed possesses secondary metabolites that inhibit virulence of pathogenic bacteria. One such compound is melatonin, which inhibits bacterial T3SS genes expression but no bactericidal effect. This is due to the mechanism that melatonin is sensed by CpxA^E48/T51^, which inhibits the phosphorylation cascade transmission from CpxA^H240^ to CpxR^D52^, resulting in the inhibition of DNA‐binding capacity of CpxR and subsequent T3SS genes expression (Figure 4I). Based on this molecular basis, we propose the use of genetic engineering to enhance melatonin to improve plant disease resistance, or the development of novel melatonin‐related biopesticides to prevent related diseases.

## 5 Materials and Methods

### 5.1 Gene Identification

The gene identifiers for *cpxA* and *cpxR* in *Pseudomonas syringae* pv. *tomato* DC3000 (*Pst* DC3000) were obtained from the KEGG database (https://www.kegg.jp/), with locus tags PSPTO_1803 and PSPTO_1806, respectively.

### 5.2 Natural Product Library Screening

The DNA sequence of *avrPto* promoter was amplified and ligated to the *pMS402* vector for construction of *avrPto promoter::LUC‐pMS402*, which was transformed into the *Pst* DC3000 strain via electroporation, yielding the *Pst* DC3000‐*LUC* strain and cultured in King's B (KB) medium to OD_600_ = 0.6 [8]. Then, the cultures were washed three times by minimal medium (MM) liquid medium and diluted to an OD_600_ = 0.4. A commercial natural product library (Catalog No.L1400, Selleck Chem, USA) containing a total of 3,048 compounds was screened to identify plant‐derived compounds with the capacity to inhibit *avrPto* expression. In brief, equal amount (50 µL) of fresh cultures were inoculated into each well of a 96‐well plate (black side wall and transparent bottom), which contained 50 µL MM liquid medium without (mock) or with 20 µM natural compounds in each well. After 12 h treatment, the treated cultures were incubated with 1 mM luciferin (Sigma, Cat # L9504) for 10 min. The LUC production was recorded by the GLO‐MAX 96 microplate luminometer (Promega).

### 5.3 Tn5‐Insertion Mutants Screening

The *Pst* DC3000 *avrPto promoter::LUC‐pMS402* strain was used to construct the Tn5‐insertion mutant library using the EZ‐Tn5<KAN> kit (BIOSEARCH, Cat # 29213). Briefly, competent cells of *Pst* DC3000‐*LUC* strain were mixed with transposon and transposase, and then were used for electroporation according to manufacturer's instructions. Subsequently, the transformed strains were spread on KB solid medium containing 25 mg L^−1^ kanamycin and 25 mg L^−1^ rifampicin for construction of mutant library. The *Pst* DC3000‐*LUC* strain and its mutant strains were cultured in KB medium to OD_600_ = 0.6. Then, the cultures were washed three times by MM liquid medium and diluted to an OD_600_ = 0.4. Equal amount (50 µL) of fresh cultures were inoculated into each well of a 96‐well plate (black side wall and transparent bottom), which contained 50 µL MM liquid medium without (mock) or with 20 µM melatonin in each well. After 12 h treatment, the cultures were incubated with 1 mM luciferin (Sigma, Cat # L9504) for 10 min. The LUC production was recorded by the GLO‐MAX 96 microplate luminometer (Promega). When mutant strain with *avrPto* expression insensitive to melatonin were screened, ME‐F/R was used as PCR amplification primer to identify the DNA flanking sequence of Tn5‐insertion site according to the manufacturer's instructions.

### 5.4 Construction of Pst DC3000 Mutants

To facilitate detection of AvrPto protein, we constructed *Pst* DC3000 strain with AvrPto‐Flag fusion protein. The *AvrPto‐Flag* gene was cloned into pK18*mobsacB* vector, and then were introduced into *Pst* DC3000 competent cells by electroporation. Single crossover strain was selected on KB solid medium containing 25 mg L^−1^ kanamycin. The screened strain was grown in 5 mL KB liquid medium for 4 h, and then was spread on KB solid medium containing 5% sucrose for mutant screen. The *Pst* DC3000 *AvrPto‐Flag* strain was used as wild‐type (WT) strain in all study.

The *ΔcpxA* and *ΔcpxR* strains were constructed using in‐frame deletion mutant method [53]. The upstream and downstream fragments of *cpxA* and *cpxR* coding regions were amplified and ligated to the *PK18mobsacB* vectors, which were introduced into WT competent cells by electroporation. The screening process of *PK18mobsacB*‐transformed strains was described above.

The *cpxA^E48A^*, *cpxA^T51A^*, *cpxA^H240A^*, and *cpxR^D52A^* strains were generated by site‐directed mutagenesis (Chiu et al., 2004). In brief, *cpxA* and *cpxR* fragments were cloned into *pMD18‐T* vectors, which were used as template to amplify *cpxA^E48A^*, *cpxA^T51A^*, *cpxA^H240A^*, and *cpxR^D52A^* mutant genes. Subsequently, the fragments were recombined into *PK18mobsacB* vectors. The reconstructed *PK18mobsacB* vectors were introduced into WT competent cells by electroporation. The screening process of *PK18mobsacB*‐transformed strains was described above.

### 5.5 Melatonin Treatment

Various genotypes of bacteria were cultured in KB liquid medium to OD_600_ = 0.6. Then, the cultures were washed three times by MM liquid medium and diluted to an OD_600_ = 0.4. This MM is used to maintain the basic growth of bacteria, simulate the infection state of pathogenic bacteria in plants, and induce the T3SS genes expression [28, 54]. Equal amount of fresh cultures without (mock) or with different concentrations of melatonin were inoculated into 50 mL centrifuge tubes or 96‐well plates for 12 h. Then, the cultures were collected for measurement.

### 5.6 Bacterial In‐Planta Experiments

*Arabidopsis thaliana* Columbia (Col‐0) ecotype and its *atsnat* mutant (SALK_020577; [25]) were obtained from the *Arabidopsis Biological Resource Center*. The tomato (*Solanum lycopersicum*) Ailsa Craig (AC), and their transgenic transgenic lines of *SlCOMT* overexpression (*SlCOMT*‐OE) and *SlCOMT* RNA‐interference (*SlCOMT*‐RNAi) were obtained form previous study [37]. Germinated seeds of different genotypes were sown into small pots containing seedling substrates in an environmentally controlled conditions: 23/20°C, 16/8 h, light/dark, for *Arabidopsis*; 30/20°C, 12/12 h, light/dark, for tomato. Hoagland's nutrient solution was watered daily to provide water and nutrients for seedling growth. About one month old seedlings were used for subsequent experiments.

Different genotypes of bacteria were cultured overnight at 28°C in KB liquid medium containing 25 mg L^−1^ rifampicin, then were resuspended with 10 mM MgCl_2_. Whole plants were sprayed with bacterial suspensions at a final concentration of 10^7^ colony‐forming units (CFU) mL^−1^. Leaves were obtained at 3 dpi for T3SS genes expression analysis by RT‐qPCR. Bacterial *in‐planta* growth was measured at 0, 3, or 5 dpi. Leaf disks (1 cm^2^) were collected and ground in sterile water. Leaf slurry were diluted to proper concentration and plated on a KB solid medium containing 25 mg L^−1^ rifampicin for bacterial count [55].

In field experiment, AC and *SlCOMT*‐OE plants were sprayed with *Pst* DC3000 bacterial suspension (10^7^ CFU mL^−1^) at 30, 60, and 90 days after planting. The resistance phenotype, photosynthetic rate, biomass, and fruit yield were measured at 130–150 days after planting. Resistance phenotype was photographed from plants representing the average level of the treatment. Photosynthetic rate was measured with an open‐flow gas exchange system *LI‐6400* (LI‐COR Biosciences, Lincoln, USA) [56]. The dry weight of the whole plant was recorded as biomass. The weight of all the fruit in a single plant was recorded as yield.

### 5.7 Real‐Time Quantitative PCR Assay

According to Minimum Information for Publication of Quantitative Real‐Time PCR Experiments guidelines [57]. The real‐time quantitative PCR (RT‐qPCR) were used for quantification of genes expression using *16S rRNA* as internal control. Bacterial total RNA was extracted for cDNA synthesis using Bacteria RNA Extraction Kit (Vazyme Biotech, China, Cat # R403‐01) and Fast King RT Kit (Vazyme Biotech, China, Cat # R312‐01), separately. RT‐qPCR was performed by a HiScript II Q RT SuperMix for qPCR (Vazyme Biotech, China, Cat # R223‐01) according to the manufacturer's instructions. Each reaction was performed in a 25 µL reaction volumes with 800 ng cDNA and 100 nM primers. The mRNA fold change was estimated by the threshold cycle (*C_t_*) values of 2^−(ΔΔ^ *^Ct^* ^)^. All reactions were conducted with three repeats.

### 5.8 Western Blot

The *Pst* DC3000 strains were cultured overnight in KB liquid medium, and after centrifugation at 10,000 × *g* to collect the cells, the pellet was washed three times with MM liquid medium, adjusted to OD_600_ = 0.4, and centrifuged again before lysing the collected cells with 200 µL of 20% SDS. Then, the bacterial lysate was centrifuged and the supernatant was collected as total protein. Total protein was separated on a 12% polyacrylamide gel electrophoresis before being transferred onto a PVDF membrane using a semidry blotting apparatus. The AvrPto‐Flag protein was analyzed by immunoblot using anti‐Flag Tag Mouse Monoclonal Antibody (Beyotime, Cat # AF519‐1). The anti‐RNAP antibodies (EnoGene, Cat # E2382083) were used as control. Protein quantification was performed by ImageJ.

### 5.9 Molecular Docking

Three‐dimensional structures of small molecular ligand (melatonin) and macromolecular receptor/sensor protein (CpxA) were separately obtained from ZINC database (https://zinc.docking.org/) and Protein Database website (https://alphafold.ebi.ac.uk/entry/Q885M8). Molecular docking of CpxA and melatonin was performed to show the potential binding sites with Auto dock Vian and Pymol software.

### 5.10 Protein Expression and Purification

The *cpxA* and *cpxR* genes were amplified from *Pst* DC300 genomic DNA and cloned into a pET‐30a and pGEX‐6P‐1 vectors using ClonExpressTM II One Step Cloning Kit (Vazyme, Nanjing). *E. coli* BL21 (DE3) cells containing pET30a‐*cpxA* and pGEX‐6P‐1‐*cpxR* were grown at 37°C in LB media in the presence of 25 mg L^−1^ ampicillin and kanamycin to an appropriate optical density and induced with 1 mM IPTG for 16 h at 16°C incubator shaker. The pET30a‐*cpxA* centrifugally collected the bacteria at 10,000 *g*, added the lysate (50 mM NaH_2_PO4, 300 mM NaCl), resuspended the bacteria, and then added DTT and PMSF with a final concentration of 1 mM for ultrasonication. Then centrifugation at 13,000 *g* for 30 min, the supernatant was collected and filtered by 0.45 µM filter. BeyoGold^TM^ His‐tag Purification Resin (Beyotime Cat # P2218) was incubated at 4°C for 5–8 h and then filtered, and add wash buffer (50 mM NaH_2_PO4, 300 mM NaCl, 2 mM imidazole) and wash three times. Elution was done with elution buffer (50 mM NaH_2_PO_4_, 300 mM NaCl, 50 mM imidazole), and the protein was collected by centrifugal column. For pGEX‐6P‐1‐*cpxR* centrifuged the bacteria at 10 000 *g* and added the lysate (140 mM NaCl, 2.7 mM KCl, 10 mM Na_2_HPO_4_, 1.8 mM KH_2_PO_4_). The bacteria were re‐suspended, and then DTT and PMSF with a final concentration of 1 mM were added for ultrasonic crushing. Then centrifugation at 13 000 g for 30 min, collect the supernatant and filter it with 0.45 µM filter. Then add BeyoGold GST‐tag Purification Resin (Beyotime Cat # P2250) and incubate at 4°C for 3–4 h. Then filter, add wash buffer(140 mM NaCl, 2.7 mM KCl, 10 mM Na_2_HPO_4_, 1.8 mM KH_2_PO_4_) and wash three times. Elution was done with elution buffer (50 mM Tris‐HCl, 10 mM GSH), and finally protein was collected by centrifugal column.

### 5.11 MicroScale Thermophoresis Assay

MicroScale Thermophoresis (MST) assay was performed using a NanoTemper Monolith NT. LabelFree instrument (NanoTemper Technologies GmbH, Germany) according to our previous description  [58] (Liu et al., 2024a). For each assay, the 50 nM CpxA or mutant protein was mixed with an equal volume of ligands (melatonin, indole, serotonin and IAA) at different serial concentrations in buffer (10 mm PBS, pH 7.4, and 137 mm NaCl). The samples were loaded into standard glass capillaries (Monolith NT.LabelFree capillaries), and thermophoresis analysis was performed (LED 40%, medium MST power) with MO. Control software (NanoTemper Technologies GmbH, Germany). For each set of binding experiments, three independent MST measurements were obtained at 360 nm. The datasets were processed with MO Affinity Analysis software (NanoTemper Technologies GmbH, Germany) [59].

### 5.12 Autophosphorylation Assay

For autophosphorylation assay, 1 µM purified CpxA‐His recombinant protein was incubated without (mock) or with treatment reagents (melatonin, indole, serotonin and IAA), and then added to 20 µL reaction buffer (50 mM Tris‐HCl pH 7.8, 25 mM NaCl, 25 mM KCl, 5 mM MgCl_2_) containing 10 µCi γ‐^32^P labeled ATP (PerkinElmer, USA) for 2–10 min. The reaction was stopped by 5 ×  SDS‐PAGE loading buffer. The phosphorylated proteins were separated by 12% SDS‐PAGE. After electrophoresis, SDS‐PAGE gels were dried with gel‐dryer (AlphaMetrix Biotech, Germany) and then exposed to a medical X‐ray film (Fujifilm. Japan) overnight. Protein quantification was performed by ImageJ.

### 5.13 Phos‐Tag Acrylamide Gel Analysis

In vitro phosphorylation assay was performed in kinase assay buffer (20 mM Tris‐HCl buffer, 100 mM NaCl, 20 mM MgCl_2_, 2 mM DTT, 10 mM ATP) using His‐fused and Strep‐fused proteins. The samples were incubated for 30 min at 30°C, and the reactions were stopped by adding 5× loading buffer and boiling for 5 min. Then, the samples were separated using 10% SDS‐PAGE, with 0.1 mM of MnCl_2_ and 0.1 mM of Phos‐Tag Acrylamide (Cat # AAL‐107, Fujifilm wako chemicals, Japan) and the proteins were detected using anti‐His antibody (SC‐8036, Santa Cruz Biotechnology, Beijing, China) and anti‐Strep antibody (Cat # AF2924, Beyotime, China) [60].

### 5.14 Transcriptome Analysis

Total RNA was extracted using RNeasy Mini Kit (Qiagen, Cat # 74104). Genomic DNA and rRNA were degraded using DNaseI (NEB, USA) and MICROB Express Kit (Ambion), separately. The mRNA was used to generate the cDNA library according to the NEB Next UltraTM II RNA Library Prep Kit protocol (NEB, USA). Subsequently, the cDNA library was sequenced using the HiSeq 2000 system (Illumina, USA) with two independent biological replicates. Clean reads were aligned to the *Pst* DC3000 reference genome (NCBI txid: 223283) using STAR, gene expression levels were quantified as transcripts per million (TPM) with StringTie, differential expression analysis was performed using DESeq2 with statistical significance adjusted via the Benjamini–Hochberg false discovery rate (FDR) correction, and genes with an adjusted *P*‐value (FDR) < 0.05 and |log_2_(fold change)| ≥ 1 were defined as significantly differentially expressed.

### 5.15 ChIP‐Seq and ChIP‐qPCR

In terms of strain construction, we first introduced a His‐tag just before the stop codon of *cpxR* to generate a *cpxR‐His* strain. Similarly, we introduced a His‐tag immediately before the stop codon of *cpxR* in the *cpxA^E48A^* and *cpxA^T51A^* mutant backgrounds, generating the *cpxA^E48A^‐His* and *cpxA^T51A^‐His* strains. The bacterial cultures were divided into two parts when their OD_600_ reached 0.6. One part was treated with 10 µM melatonin for 12 h, while the other was not for the same duration. Subsequently, 1% formaldehyde was added for cross‐linking, then quenched by 0.5 M glycine. Cross‐linked cells were harvested, lysed and ultrasonicated. Cell debris was pelleted, and the supernatant was used for IP of protein‐DNA complexes with anti‐His antibody (SC‐8036, Santa Cruz Biotechnology, Beijing, China) overnight at 4°C. Magnetic protein A/G beads were added for additional incubation at 4°C. The eluted samples were reversed cross‐linked and incubated with RNaseA and proteinase K solution overnight at 45°C. The WT strain was used as negative control. For ChIP‐Seq assay, immunoprecipitated DNA was used to construct sequencing libraries following the protocol provided by the I NEXTFLEX ChIP‐Seq Library Prep Kit for Illumina Sequencing (NOVA‐5143‐02, Bioo Scientific) and sequenced on Illumina Novaseq 6000 with PE 150 method. ChIP‐Seq peaks were called using MACS2 [61] with the callpeak module. Parameters were set as: ‐f BAMPE for paired‐end reads, ‐g 6.5e6 (effective genome size of *Pst* DC3000), and ‐q 0.05 (FDR cutoff). Wild‐type input DNA was used as the background control. For ChIP‐qPCR assay, immunoprecipitated DNA was purified using a PCR Purification Kit (Qiagen, Cat # 28104). The relative enrichment of promoters of *avrPto*, *hrpL*, and *16S rRNA* (negative control) was examined by RT‐qPCR as previous description.

### 5.16 Electrophoretic Mobility Shift Assay

The coding sequence of *cpxR* was cloned into pGEX‐6P‐1 vector for fusion with GST tag. The CpxR‐GST recombinant protein were expressed in *E. coli* strain after induction with 1 mM IPTG for 10 h at 28°C. The motif fragments in promoters of *avrPto* and *hrpL* were synthesized and labeled with 5‐FAM (Fluorescein) probes. 0.08 pmol labeled probes were incubated with 2.5 mg purified recombinant proteins in the 20 mL binding system [20 mM Tris‐HCl, pH 7.5, 100 mM NaCl, 2 mM MgCl_2_, 1 mM DTT, 10% (v/v) glycerol] at 25°C for 30 min. Only application of probe and co‐application of probe and GST recombinant protein (empty vector) were used as the negative control. For competition assay, unlabeled competitors were added in the reaction at 10 and 100‐fold. Electrophoresis was performed with native 8% polyacrylamide gels in 0.5× Tris‐borate‐EDTA for 90 min at 140 V in the dark at 4°C. The gels were scanned using a Tanon (5200 Multi) imaging system.

### 5.17 Melatonin Measurement

Four hundred milligram of frozen plant samples were ground to a powder in liquid nitrogen using a mortar and pestle and extracted with 4 mL of 50 mM sodium phosphate buffer (pH 7.9). After centrifugation for 5 min at 13 500 × *g*, the supernatants were added with 80 µL of 0.1 m KOH and then followed by 2 mL chloroform extraction. The chloroform fractions were mixed with 0.5 mL of 0.45 M sodium borate buffer (pH 10.0) and then centrifuged as above. The samples were then evaporated and dissolved in 0.1 mL of water, and 10 µL aliquots were subjected to high‐performance liquid chromatography (HPLC) with a fluorescence detector system (Waters). The samples were separated on an Atlantis C18 column (Waters; 3.9 × 150 mm) with an isocratic elution profile of 37% (v/v) MeOH in water at a flow rate of 0.15 mL min^−1^. Melatonin was detected at 280 nm excitation and 348 nm emission.

### 5.18 Other Information

Source and raw data of RNA‐Seq and ChIP‐Seq have been provided in Supplementary Dataset and Sequence Read Archive database (PRJNA218897). Information of gene ID, primers, and vectors are listed in Supplementary Dataset.

### 5.19 Statistical Analysis

All quantitative data were derived from at least three independent biological replicates and are presented as the mean ± standard deviation (SD). The sample size (n) for each experiment, representing the number of biological replicates, is specified in the corresponding figure legends. Statistical analysis was performed using GraphPad Prism software (version 8.0; GraphPad Software, San Diego, CA, USA). To assess significant differences between treatment groups and their corresponding controls, one‐ or two‐way Analysis of Variance (ANOVA) with Sidak's or Dunnett's multiple comparison test was conducted, as indicated in the figure legends. All ANOVA tests were two‐sided and met the assumptions of normality and homogeneity of variance. The significance level (alpha) was set at 0.05. *P*‐values are denoted as follows: ^ns^ *P* > 0.05 (not significant), *P < 0.05, **P < 0.01, ***P < 0.001, ****P < 0.0001; or different letters indicate significant differences at *P* < 0.05.

## Author Contributions

Conceptualization: B.G. and J.W. Methodology: J.W., W.L., B.C., D.Z., C.W., X.H., T.N., and B.G. Investigation: B.G. and J.W. Visualization: W.L., B.C., and D.Z. Supervision: B.G., T.N., and X.H. Writing –original draft: B.G. and J.W. Writing –review and editing: T.N., and X.H.

## Funding

National Natural Science Foundation of China (32573008 and 32272697), Taishan Scholars Program (tsqn202306139), National Key R&D Program of China (2023YFD200140403), Shandong Province Modern Agricultural Technology System (SDAIT‐25‐02), Shandong Provincial “811” Project of First‐class Discipline Construction.

## Conflicts of Interest

The authors declare no conflicts of interest.

## Supporting information




**Supporting File 1**: advs74806‐sup‐0001‐SuppMat.pdf.

**Supporting File 2**: advs74806‐sup‐0002‐DataFile.xlsx.

## Data Availability

The data that supports the findings of this study are available in the supplementary material of this article.
