# Supplementary material for: Plant‐Derived Melatonin Inhibits Bacterial Virulence via CpxA/R Two‐Component System
Source: Adv Sci (Weinh). 2026 Mar 12;13(32):e74806. doi: 10.1002/advs.74806 (PMC13252655; doi:10.1002/advs.74806)
Supplement: Supplementary file 1 — Supporting File 1: advs74806‐sup‐0001‐SuppMat.pdf. [file ADVS-13-e74806-s002.pdf]

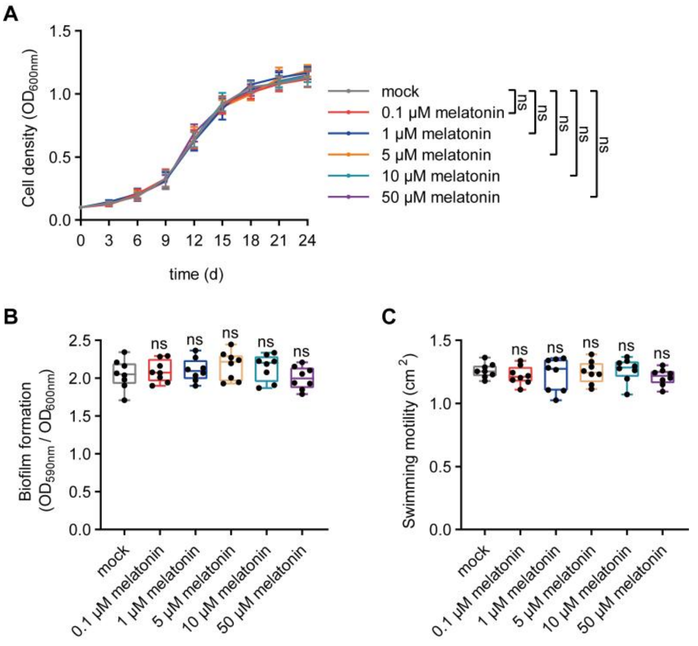


**Supplementary Figure S1. Physiological effect of melatonin on *Pst* DC3000. (A)** Bacterial growth analysis. *Pst* DC3000 was cultured in King's B (KB) liquid medium to $OD_{600}$ = 0.6, and then was washed with KB liquid medium and adjusted to $OD_{600}$ = 0.01 with 0-50 μM melatonin. Every 20 mL cell suspension of each treatment was cultured in 50 mL tube at 28°C and 180 rpm. The cell density ($OD_{600}$) was measured for growth analysis every three hours. Data represents means ± SD (n = 8, two-way ANOVA with Dunnett's multiple comparison test; $^{ns}P > 0.05$). **(B)** Bacterial biofilm formation. *Pst* DC3000 was cultured in KB liquid medium to $OD_{600}$ = 0.6, and then was washed with minimal medium (MM) liquid medium and adjusted to $OD_{600}$ = 0.4 with 0-50 μM melatonin. Every 200 μL cell suspension of each treatment was cultured in a well of 96-well plates for 24 h to measure the $OD_{600}$. Then, cells were stained with 0.1% crystal violet (CV) for 30 min. The planktonic cells were washed away with water. The CV-stained bound cells were air-dried for 1 h and then dissolved in 95% ethanol to measure the $OD_{590}$. Biofilm formation was calculated as the ratio of $OD_{590}/OD_{600}$. Data represents means ± SD (n = 8, one-way ANOVA with Dunnett's multiple comparison test; $^{ns}P > 0.05$) **(C)** Bacterial swimming motility. *Pst* DC3000 was cultured in KB liquid medium to $OD_{600}$ = 0.6, and then was washed with MM liquid medium and adjusted to $OD_{600}$ = 0.4. Equivalent bacteria was inoculated on MM solid medium containing 0.3% agar with 0-50 μM melatonin. The colony diameter was measured for swimming motility at 48 h after treatment. Data represents means ± SD (n = 8, one-way ANOVA with Dunnett's multiple comparison test; $^{ns}P >$ 0.05).

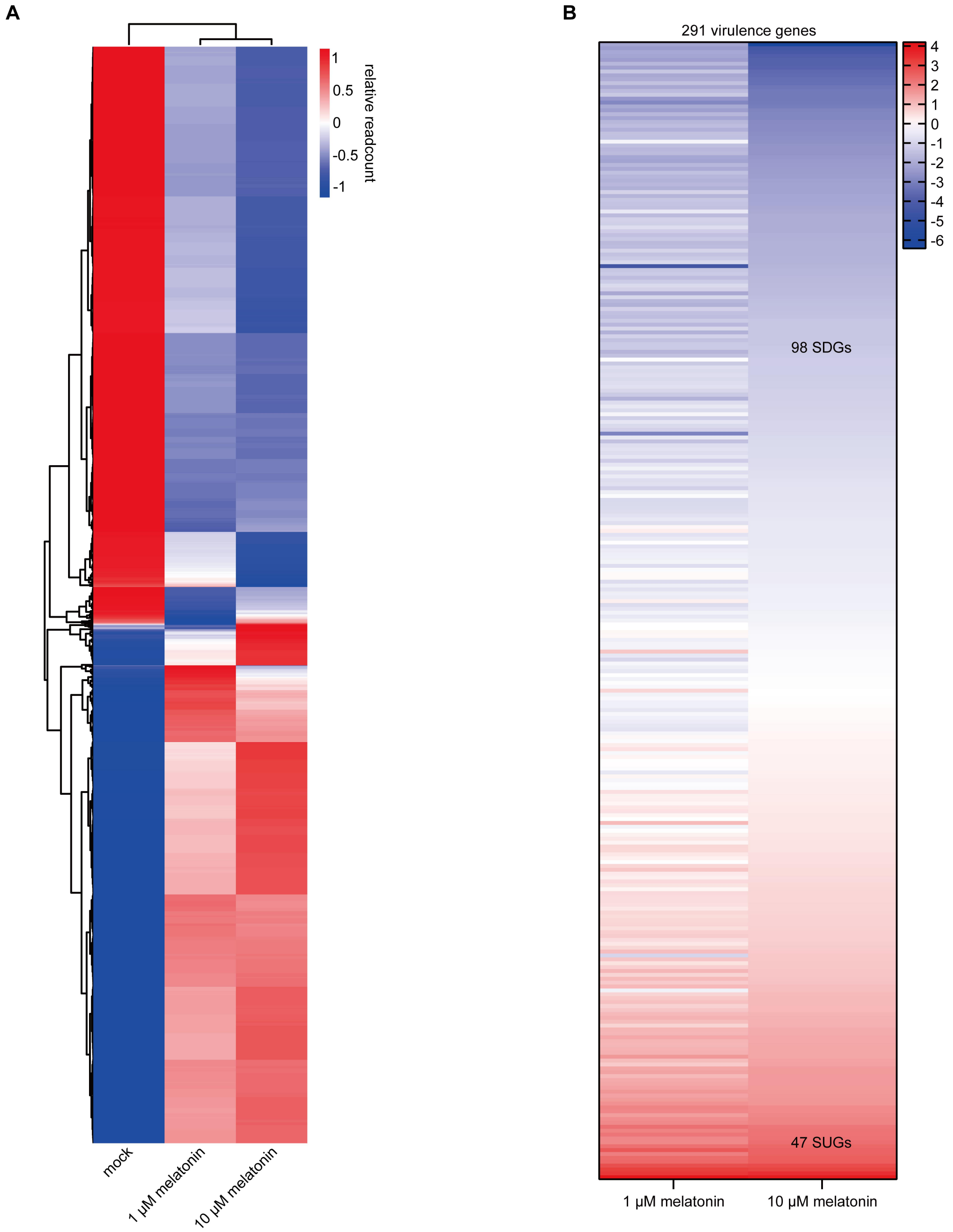


**Supplementary Figure S2. Transcriptome analysis of *Pst* DC3000 response to melatonin. (A)** Hierarchical clustering analysis of differentially expressed genes (DEGs) in RNA-Seq. **(B)** Gene expression pattern of 291 virulence genes in RNA-Seq. Gene expression levels for the 10 μM melatonin treatment group were sorted from low to high, revealing a set of 98 SDGs and 47 SUGs.

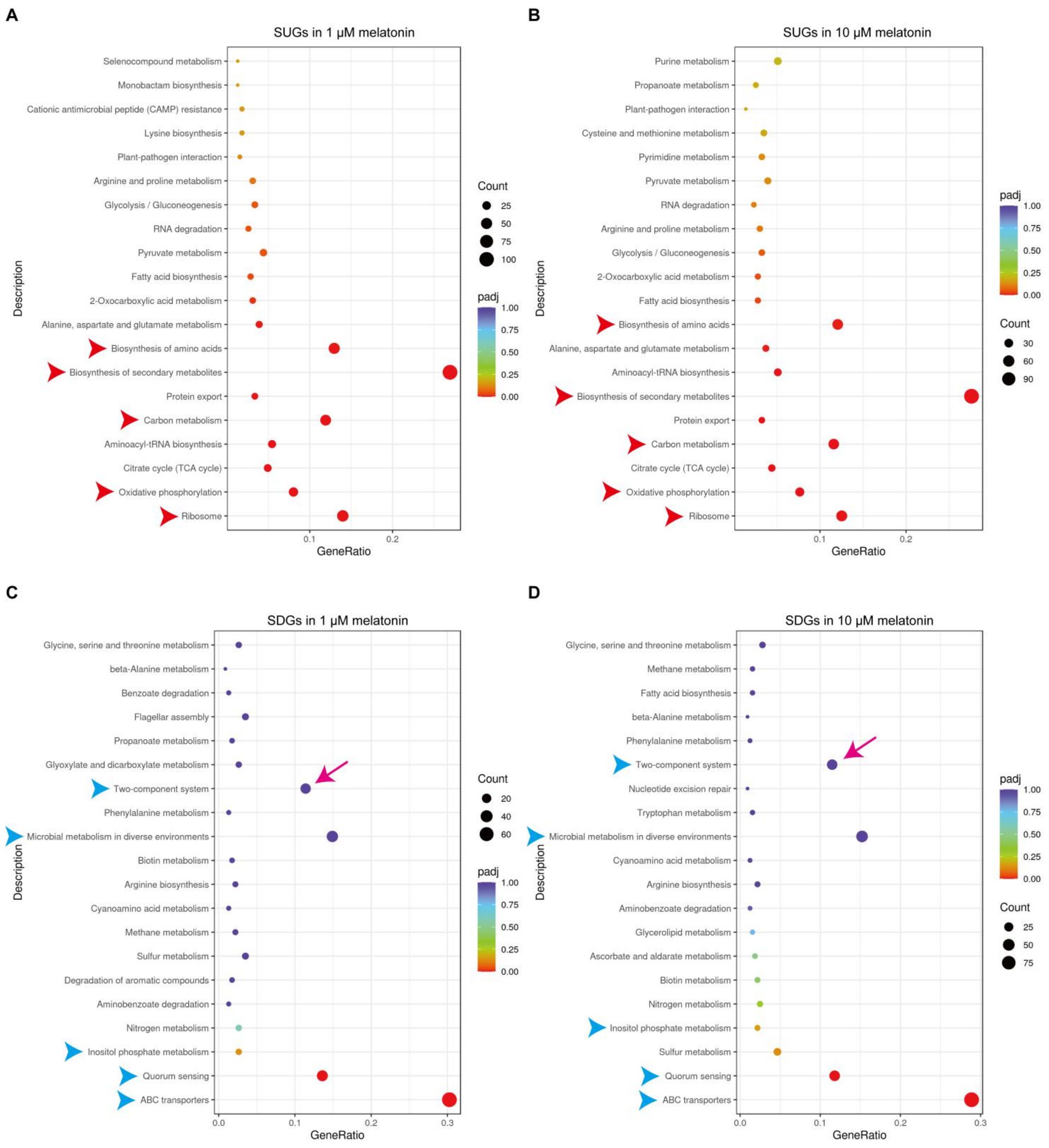


**Supplementary Figure S3. KEGG analysis of the transcriptome. (A, B)** KEGG of SUGs in "1 μM melatonin" and "10 μM melatonin" groups. Red marks indicate the top five common KEGG in both groups. **(C, D)** KEGG of SDGs in "1 μM melatonin" and "10 μM melatonin" groups. Blue marks indicate the top five common KEGG in both groups.

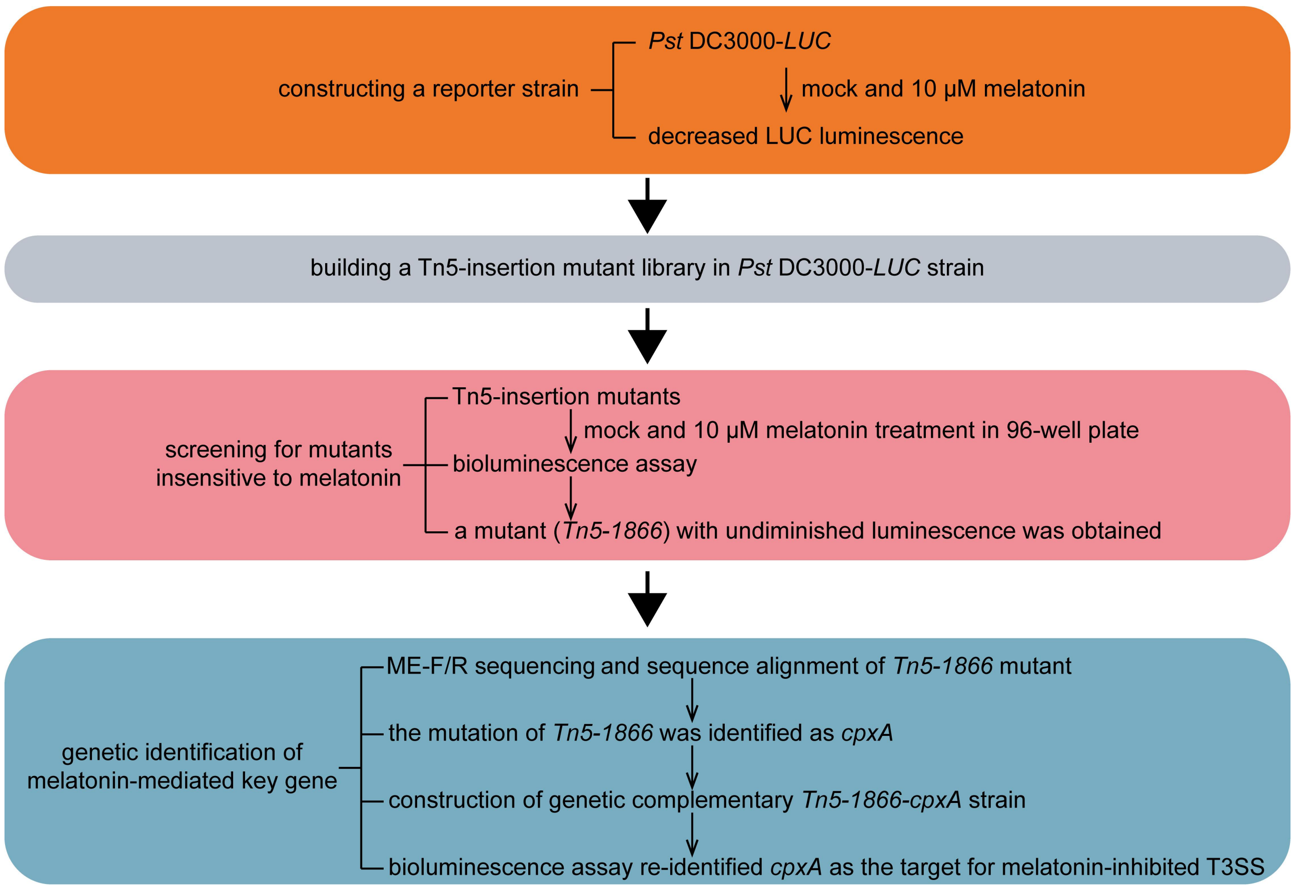


**Supplementary Figure S4. Screening the target of melatonin in inhibiting T3SS.** Flow chart for identification process: The *Pst* DC3000 *avrPto promoter::LUC-pMS402* strain was used to construct the Tn5-insertion mutant library using the EZ-Tn5<KAN> kit (BIOSEARCH, Cat # 29213). Briefly, competent cells of *Pst* DC3000-*LUC* strain were mixed with transposon and transposase, and then were used for electroporation according to manufacturer's instructions. Subsequently, the transformed strains were spread on KB solid medium containing 25 mg $L^{-1}$ kanamycin and 25 mg $L^{-1}$ rifampicin for construction of mutant library. The *Pst* DC3000-*LUC* strain and its mutant strains were cultured in KB medium to $OD_{600}$ = 0.6. Then, the cultures were washed three times by MM liquid medium and diluted to an $OD_{600}$ = 0.4. Equal amount (50 μL) of fresh cultures were inoculated into each well of a 96-well plate (black side wall and transparent bottom), which contained 50 μL MM liquid medium without (mock) or with 20 μM melatonin in each well. After 12 h treatment, the cultures were incubated with 1 mM luciferin (Sigma, Cat # L9504) for 10 min. The LUC production was recorded by the GLO-MAX 96 microplate luminometer (Promega). When mutant strain with *avrPto* expression insensitive to melatonin were screened, ME-F/R was used as PCR amplification primer to identify the DNA flanking sequence of Tn5-insertion site according to the manufacturer's instructions.

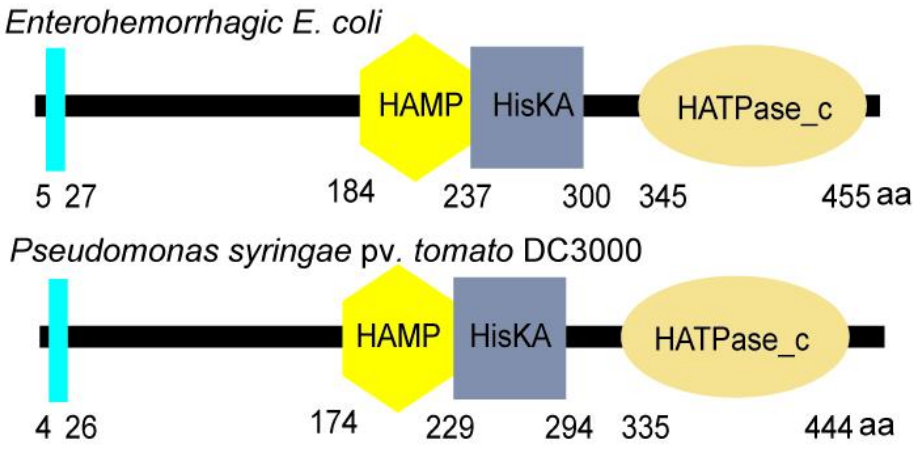


**Supplementary Figure S5. Protein structures of CpxA from *Escherichia coli* (WP_09749954.1) and *Pst* DC3000 (PSPTO_1803).** CpxA contains four conserved functional domains: a Per-Arnt-Sim (PAS) domain, a Histidine kinases, Adenylyl cyclases, Methyl-binding proteins, and Phosphatases (HAMP) domain, a His Kinase A (HisKA) phosphoacceptor domain, and a Histidine kinase-like ATPase (HATPase_C) domain.

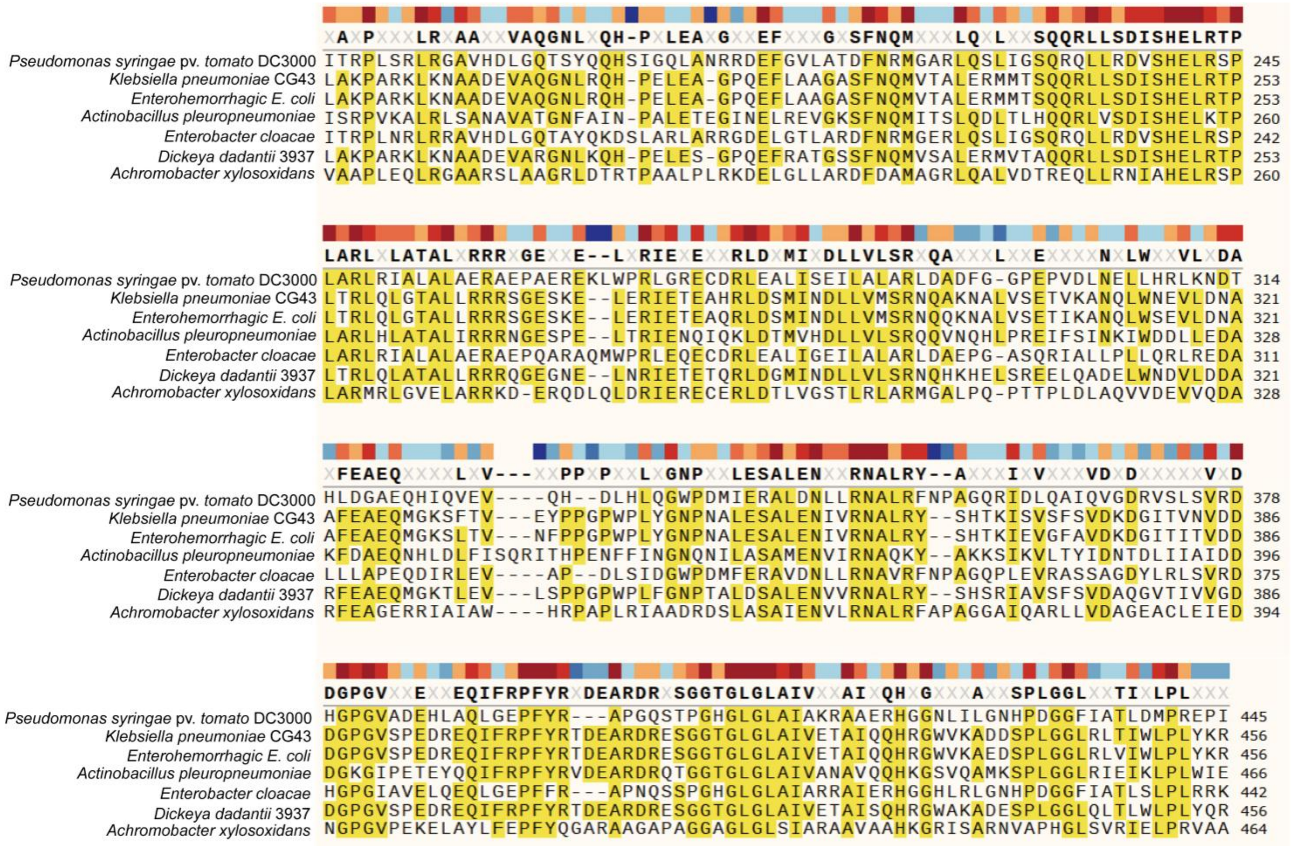

**Supplementary Figure S6. Conservation analysis of putative CpxA protein sequences.** To evaluate the conservation of the putative CpxA protein in *Pst* DC3000 relative to other bacteria, a protein sequence alignment was performed using SnapGene software across six bacterial strains with reported CpxA.

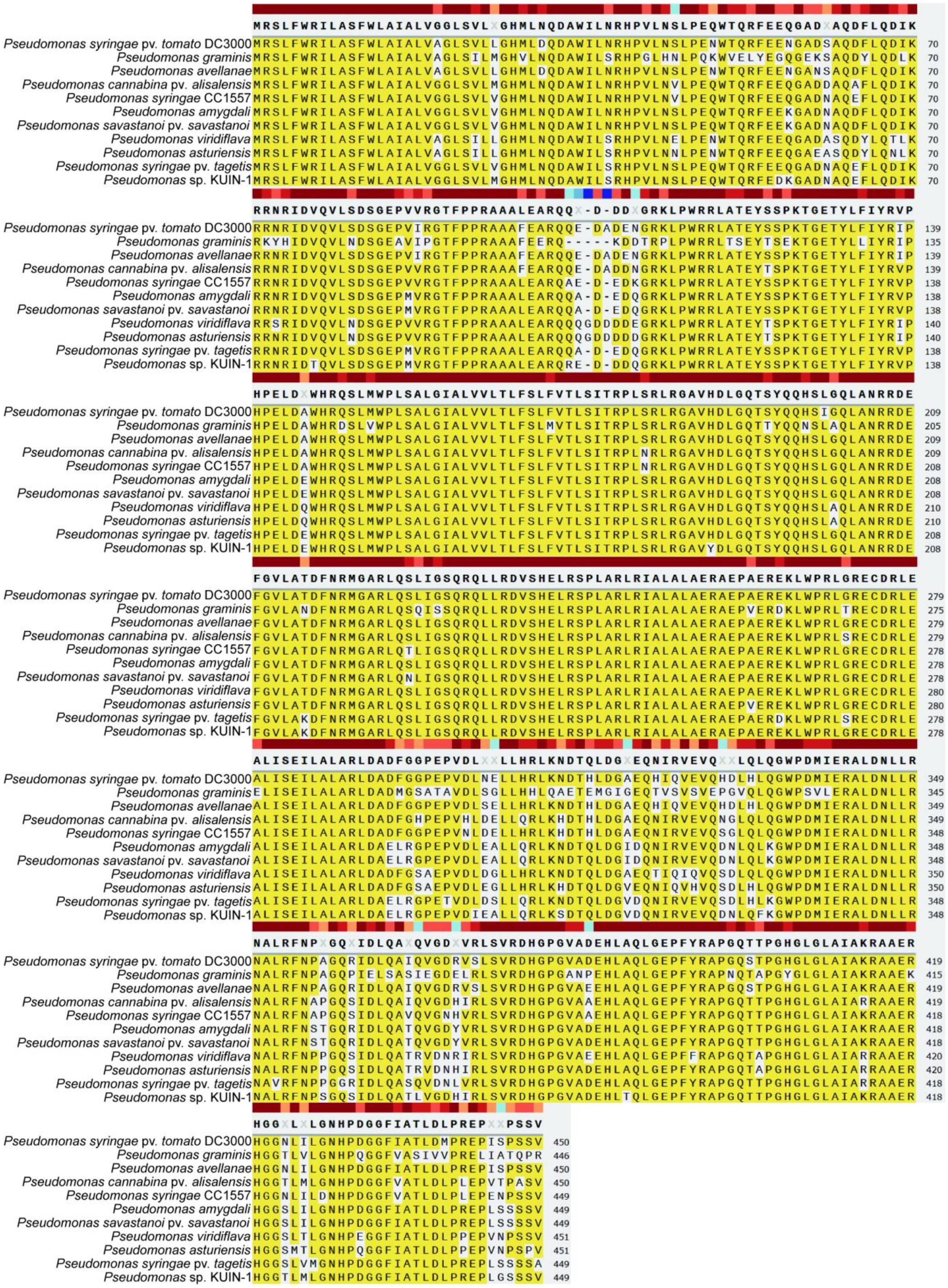

**Supplementary Figure S7. Conservation analysis of putative CpxA protein sequences across *Pseudomonas* species.** To evaluate the conservation of the putative CpxA protein in *Pst* DC3000 relative to other *Pseudomonas* species, a protein sequence alignment was performed using SnapGene software across ten bacterial strains with reported CpxA.

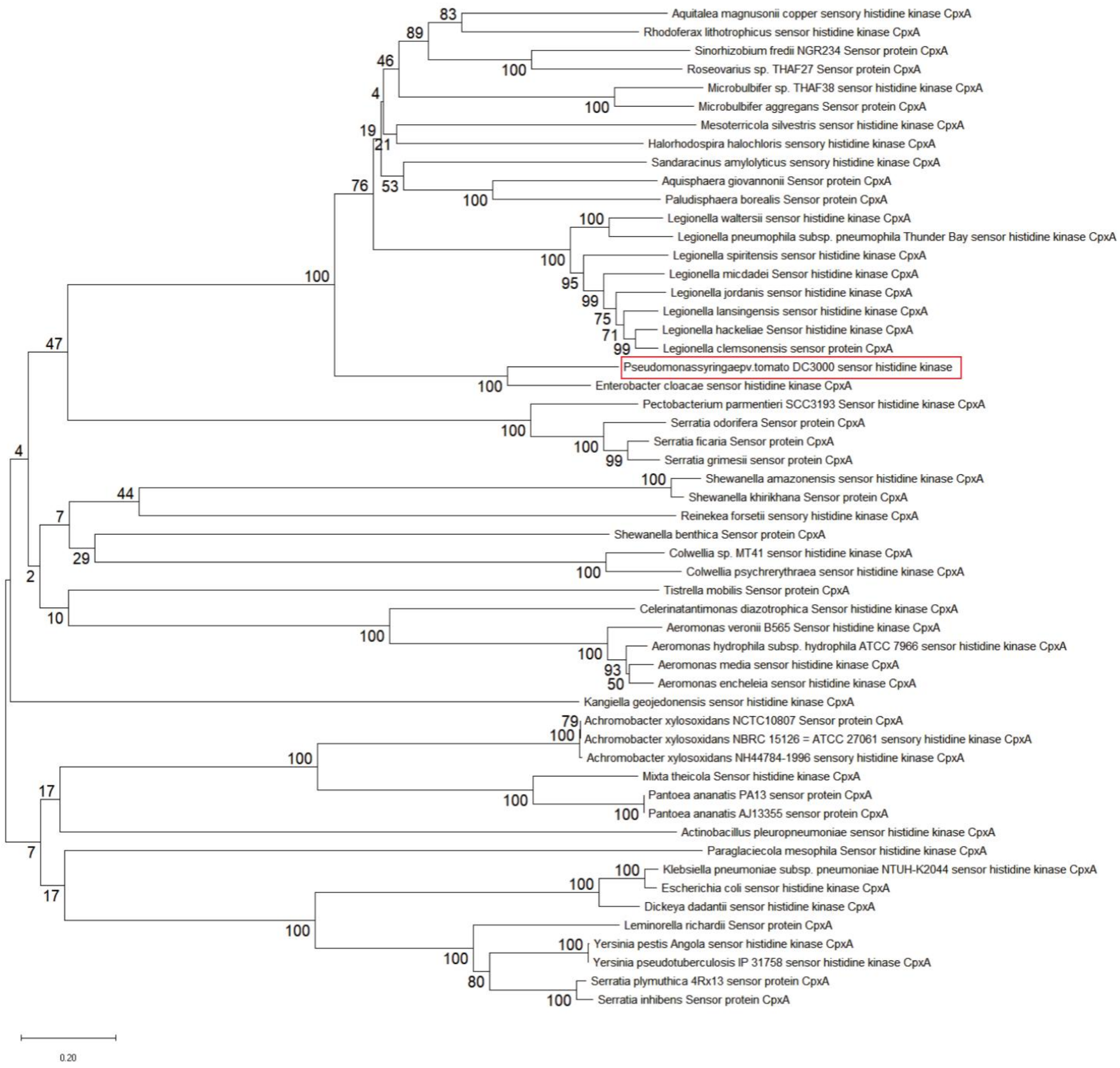


**Supplementary Figure S8. The phylogenetic tree of putative CpxA proteins.** A phylogenetic tree of putative CpxA proteins sequences from 55 bacteria was constructed using the neighbor-joining method in MEGA 11 software with 1000 bootstrap replicates, illustrating the evolutionary relationships of the putative histidine kinase CpxA across diverse bacterial genera. Bootstrap support values are displayed on the branches, and a scale bar indicating the amino acid substitution rate is provided.

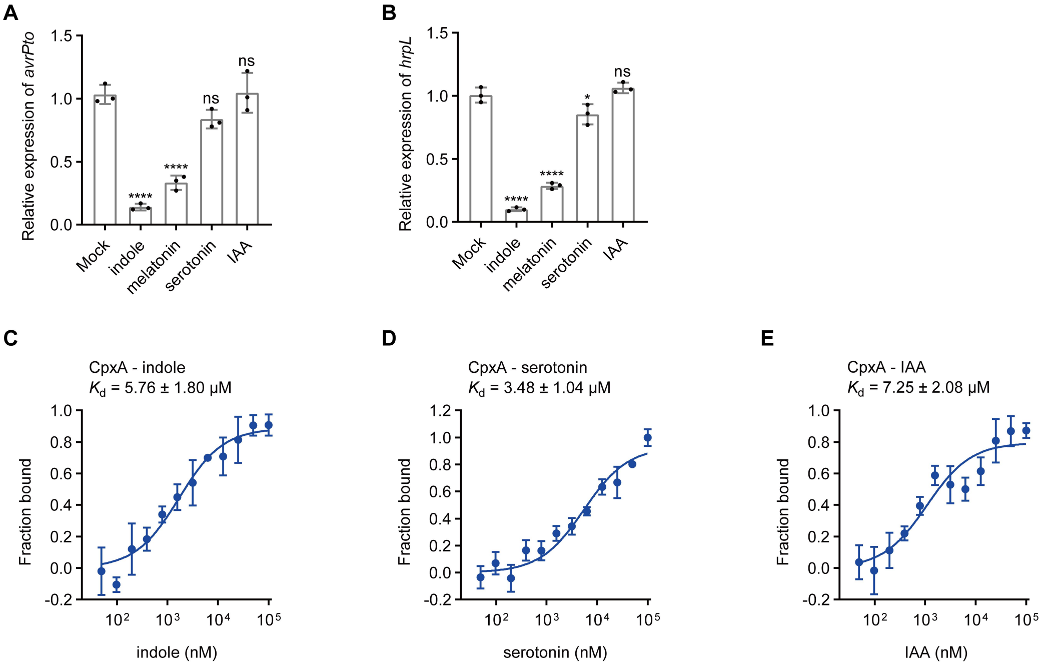


**Supplementary Figure S9. Effects of melatonin analogues on *avrPto* and *hrpL* expression and on CpxA binding. (A, B)** Response of *avrPto* and *hrpL* to indole, melatonin, serotonin, and IAA. WT strains were incubated without or with 10 μM drugs in MM liquid medium for 12 h, and then were used for RT-qPCR assay. Data of RT-qPCR represents means ± SD (n = 3, one-way ANOVA with Dunnett's multiple comparison test; $^{ns}P > 0.05$, $^{*}P < 0.05$, $^{****}P < 0.0001$). **(C-E)** Effects of indole, serotonin, and IAA on CpxA binding. Data represents means ± SD (n = 3).

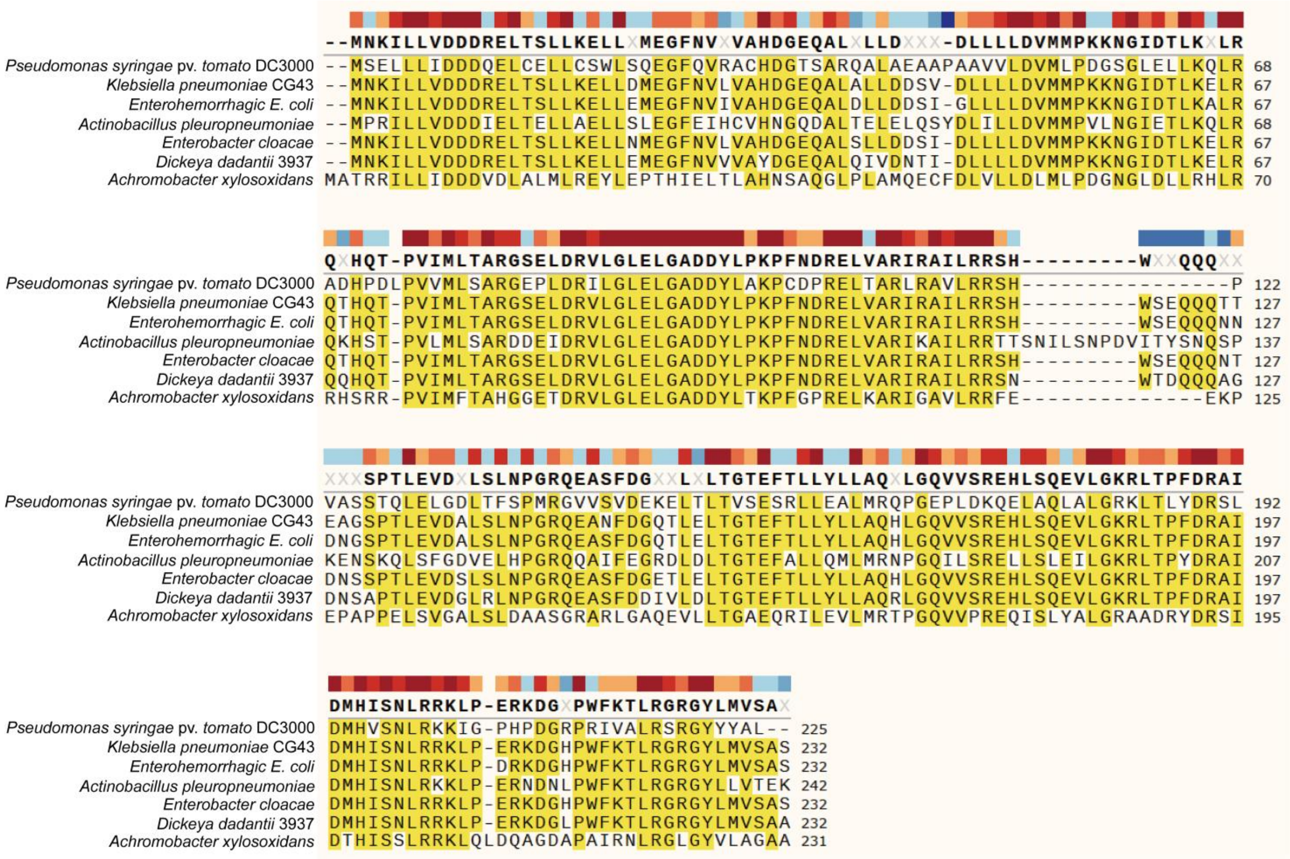

**Supplementary Figure S10. Conservation analysis of putative CpxR protein sequences.** To evaluate the conservation of the putative CpxR protein in *Pst* DC3000 relative to other bacteria, a protein sequence alignment was performed using SnapGene software across six bacterial strains with reported CpxR.

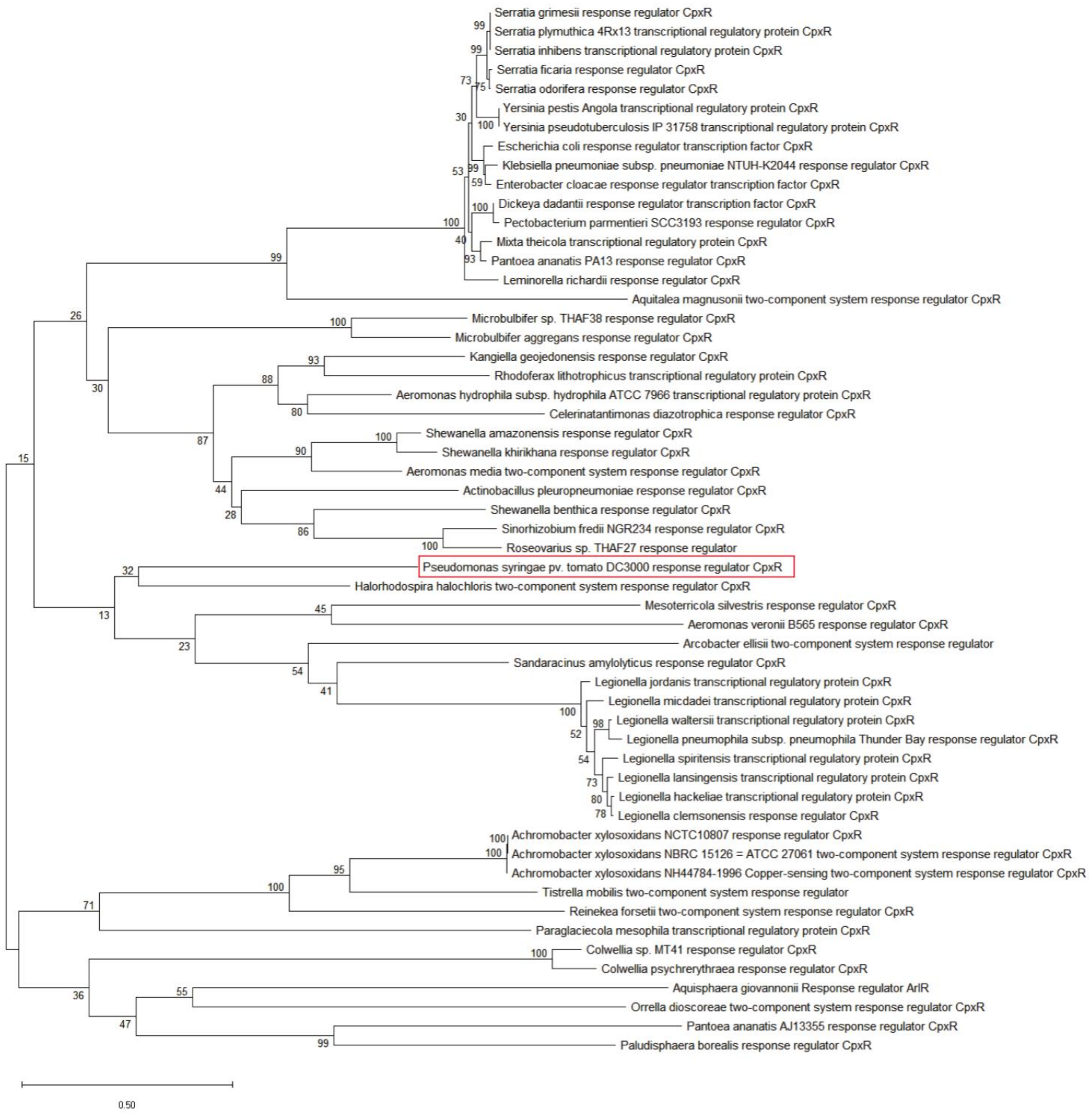


**Supplementary Figure S11. The phylogenetic tree of putative CpxR protein.** A phylogenetic tree of putative CpxR protein sequences from 55 bacteria was constructed using the neighbor-joining method in MEGA 11 software with 1000 bootstrap replicates, illustrating the evolutionary relationships of the putative CpxR protein across diverse bacterial genera. Bootstrap support values are displayed on the branches, and a scale bar indicating the amino acid substitution rate is provided.

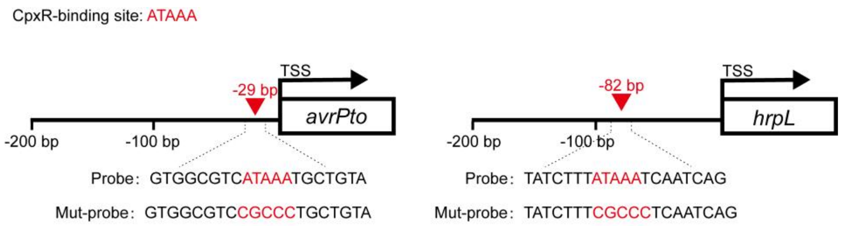


**Supplementary Figure S12. Characterization of CpxR-binding sites and design of EMSA probes for the *avrPto* and *hrpL* promoters.**

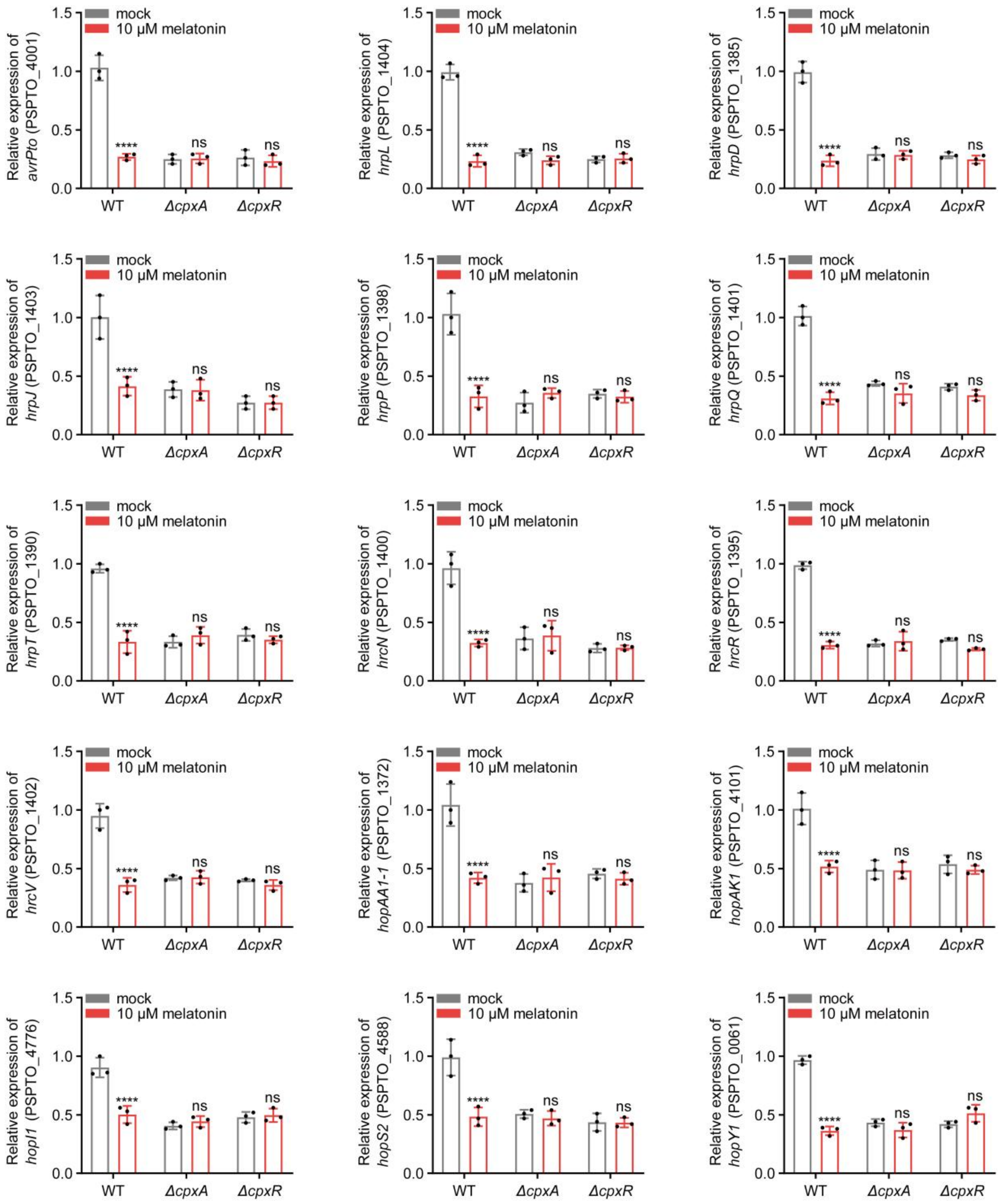


**Supplementary Figure S13. Data cross-referenced with Fig. 3G.** Melatonin inhibits the expression of core T3SS genes (intersection of Venn diagram) via the CpxA/R system. WT, *ΔcpxA*, and *ΔcpxR* strains were incubated without or with 10 μM melatonin in MM liquid medium for 12 h, and then were used for RT-qPCR assay. Data of RT-qPCR represents means ± SD (n = 3, two-way ANOVA with Sidak's multiple comparison test, $^{ns}P > 0.05$, $^{****}P < 0.0001$).

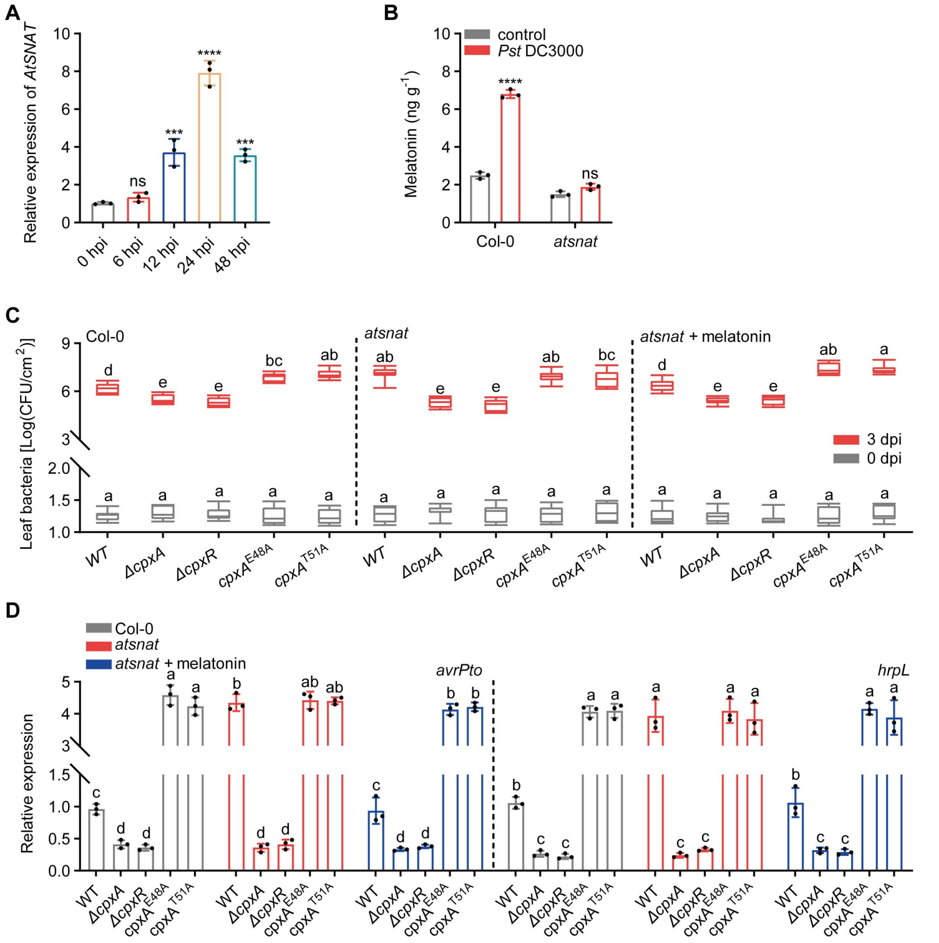


**Supplementary Figure S14. Data cross-referenced with Fig. 4A-D. (A)** Transcriptional response of *AtSNAT* to *Pst* DC3000 infection. Col-0 plants were sprayed with *Pst* DC3000 suspension ($10^7$ CFU $mL^{-1}$). Leaves were harvested for RT-qPCR assay at 0-48 hpi. Data represents means ± SD (n = 3, one-way ANOVA with Dunnett's multiple comparison test; $^{ns}P > 0.05$, $^{***}P < 0.001$, $^{****}P < 0.0001$). **(B)** Melatonin contents. Col-0 and *atsnat* were sprayed without or with *Pst* DC3000 suspension ($10^7$ CFU $mL^{-1}$). Leaves were harvested for melatonin measurement at 24 hpi. Data represents means ± SD (n = 3, two-way ANOVA with Sidak's multiple comparison test; $^{ns}P > 0.05$, $^{****}P < 0.0001$). **(C, D)** Bacterial growth and T3SS genes (*avrPto* and *hrpL*) expression in *Arabidopsis* leaves. Plants of Col-0 and *atsnat* were sprayed with bacterial suspension of WT, *ΔcpxA*, *ΔcpxR*, $cpxA^{E48A}$, and $cpxA^{T51A}$ strains ($10^7$ CFU $mL^{-1}$). For exogenous treatment, 10 μM melatonin was applied to the *atsnat* mutants every day. Bacterial growth was measured at 0, 3, or 5 dpi; boxplots show median with 0.25 and 0.75 quartiles, whiskers represent values from minimum to maximum (n = 8, one-way ANOVA with Dunnett's multiple comparison test; different letters indicate significant differences at $P < 0.05$). RT-qPCR were performed at 3 dpi; data represents means ± SD (n = 3, one-way ANOVA with Dunnett's multiple comparison test; different letters indicate significant differences at $P < 0.05$).
